# Computational Oncology of Chemotaxis-Driven Tumour–Immune Spatial Patterning and Stability

**DOI:** 10.3390/bioengineering13080952

**Published:** 2026-08-21

**Authors:** Zonghao Liu, Jiguang Yu, Louis Shuo Wang, Lei Su, Ye Liang, Yang Du, Jingfeng Liu

**Affiliations:** 1 Department of Hepatopancreatobiliary Surgery, Clinical Oncology School, Fujian Medical University, Fuzhou 350014, China; liuzonghao42@gmail.com; 2 College of Engineering, Boston University, Boston, MA 02215, USA; jyu678@bu.edu; 3 Department of Mathematics, Northeastern University, Boston, MA 02115, USA; 4 Institute of Automation, Chinese Academy of Sciences, Beijing 100190, China; sulei2023@ia.ac.cn (L.S.); yang.du@ia.ac.cn (Y.D.); 5 University of Chinese Academy of Sciences, Beijing 100190, China; 6 Department of Industrial and Systems Engineering, The University of Iowa, Iowa City, IA 52242, USA; ye-liang@uiowa.edu; 7 Key Laboratory of Cancer Metabolism, National Health Commission of the People’s Republic of China (NHC), Fuzhou 350014, China; 8 Fujian Key Laboratory of Advanced Technology for Cancer Screening and Early Diagnosis, Fuzhou 350014, China

**Keywords:** computational oncology, tumour–immune–chemokine dynamics, reaction–diffusion–chemotaxis system, tumour invasion threshold, linear stability, dispersion relation, finite-wavelength oscillatory instability, pattern formation, finite-volume method, sensitivity analysis

## Abstract

We develop a reaction–diffusion–chemotaxis model for spatial tumour–immune–chemokine dynamics that couples logistic tumour growth, immune-mediated killing, chemokine-dependent immune recruitment, chemotactic migration, and signal production. For the non-dimensional system, we establish local classical solvability, nonnegativity, a uniform tumour-density bound, and global mass estimates for the immune and chemokine components. The tumour-free equilibrium is stable precisely when the baseline immune-control index satisfies $\sigma_{0}/\delta >1$, whereas positive homogeneous coexistence is characterized by a scalar nonlinear equation. Linearization in the Neumann Laplacian eigenbasis yields a mode-dependent cubic dispersion relation, showing that chemotaxis does not alter the tumour-invasion threshold but can destabilize homogeneous coexistence through a finite-wavelength oscillatory instability above a critical sensitivity $\xi_{c}$. A conservative finite-volume discretization with upwind chemotactic fluxes and implicit backward differentiation formula time integration is used to test these predictions. Numerical experiments recover the analytical equilibria and growth rates, identify the dominant unstable mode, reproduce the transition to spatial heterogeneity, and quantify the effects of immune recruitment, decay, and diffusion on the stability boundary. Grid-refinement, mass-balance, residual, and nonnegativity diagnostics support the computational reliability of the results.

## 1. Introduction

### 1.1. Motivation: Computational Tumour Modelling

Cancer progression is governed by interacting processes across molecular, cellular, and tissue scales [1,2]. At the molecular scale, cytokines, chemokines, growth factors, and metabolic signals regulate proliferation, death, migration, and immune activation [3]. At the cellular scale, tumour cells, immune effector cells, stromal cells, and endothelial cells interact through direct contact and through diffusible mediators [4,5,6]. At the tissue scale, these local interactions produce spatially heterogeneous patterns of tumour expansion, immune infiltration, immune exclusion, necrosis, invasion fronts, and treatment response [7,8]. Therefore, tumour progression is not only a temporal growth process; it is also a spatially structured dynamical process.

Spatial mathematical models are particularly useful for describing these mechanisms because they connect local cell–signal interactions with macroscopic tumour behaviour. Continuum models based on reaction–diffusion equations describe cell proliferation, death, signal production, degradation, and passive spreading [9]. Chemotaxis terms describe directed cell migration along chemical gradients, which is especially relevant when immune cells respond to chemokine fields generated inside the tumour microenvironment [10]. In this way, reaction–diffusion–chemotaxis systems provide a mechanistic framework for studying how microscopic signalling and cell movement generate macroscopic invasion, immune infiltration, immune exclusion, or coexistence patterns [11,12,13,14,15,16,17].

From a computational viewpoint, such models also provide a bridge between mathematical analysis and numerical simulation. A biologically meaningful tumour model should preserve nonnegativity of cell densities and chemical concentrations, respect no-flux tissue boundaries when the model is posed on a closed tissue region, and produce interpretable stability or instability criteria. These requirements are not merely technical. Negative cell densities are biologically meaningless, artificial boundary fluxes may distort tumour growth or immune infiltration, and unstable numerical schemes may create patterns that are artifacts of the discretization rather than consequences of the model.

This work contributes to the development of mathematically analyzable and numerically reliable partial-differential-equation (PDE) models for tumour-related phenomena. The emphasis is not only on simulating spatial tumour–immune dynamics, but also on identifying stability thresholds and designing numerical diagnostics that preserve the biological constraints of nonnegativity and no-flux tissue boundaries. The resulting framework is intended to support mechanistic investigation of how chemokine-mediated immune recruitment and chemotactic migration shape tumour–immune spatial organization.

### 1.2. Biological Setting: Tumour–Immune–Chemokine Interactions

We consider a spatial tumour microenvironment represented by a bounded tissue domain $\Omega \subset R^{d}$, where d=1,2, or 3 depending on the application. The model contains three primary state variables:

- T(t,x) is tumour-cell density.
- E(t,x) is immune effector-cell density.
- A(t,x) is chemokine concentration.

Here t≥0 denotes time and x∈Ω denotes spatial position. The variable *T* represents the local density of proliferating tumour cells. The variable *E* represents immune effector cells, such as cytotoxic lymphocytes or other anti-tumour immune populations. The variable *A* represents a chemokine or aggregate chemotactic signal that promotes immune recruitment and guides immune cell movement.

**Figure 1:**
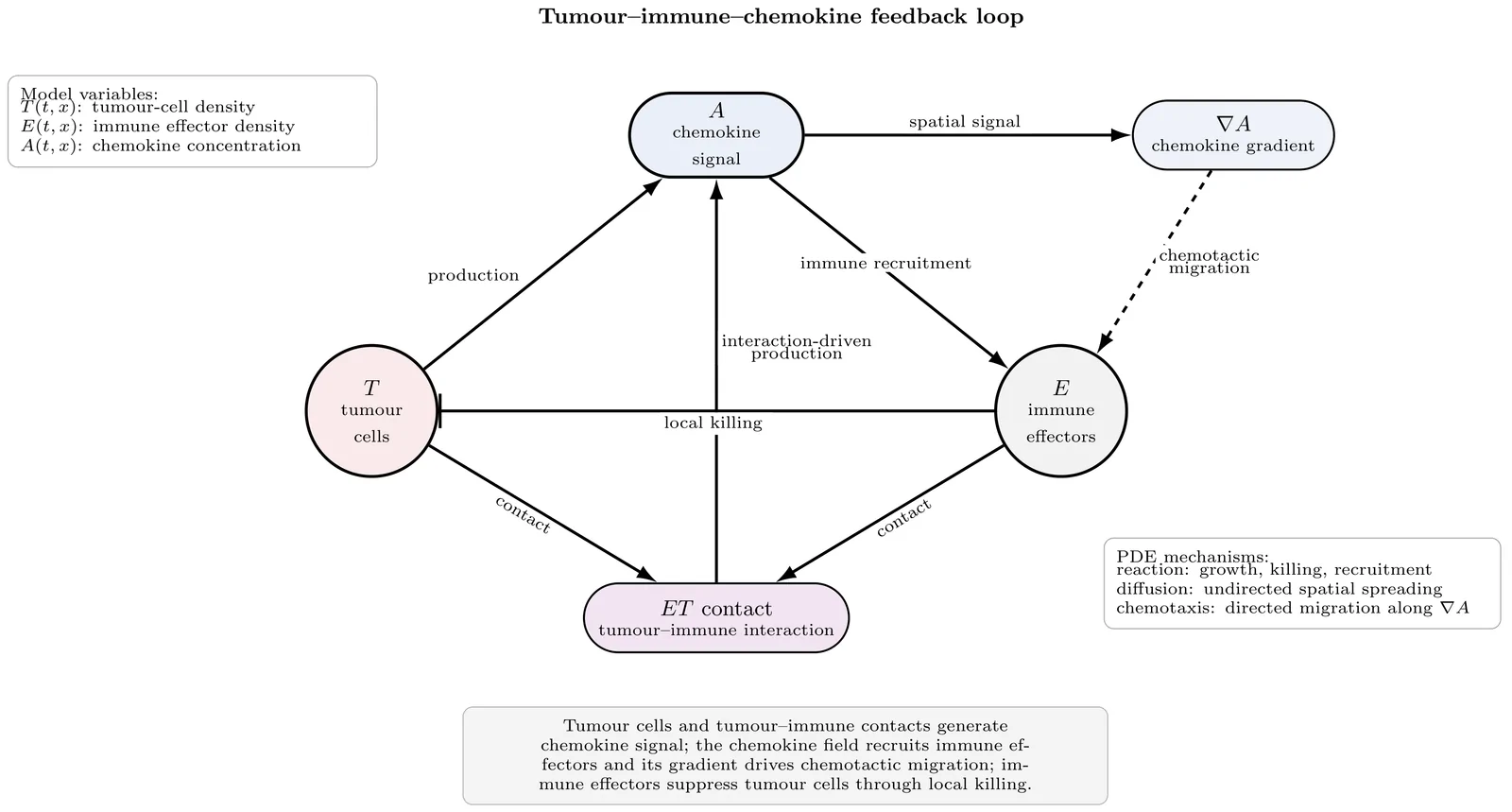
Tumour–immune–chemokine feedback loop used in the computational oncology model. Tumour cells (*T*) produce chemokine (*A*), while tumour–immune contacts (ET) provide an additional chemokine source. The chemokine field recruits immune effector cells (*E*), and its spatial gradient (∇A) drives chemotactic immune migration. Immune effectors suppress tumour cells through local killing. In the diagram, regular arrows indicate biological reactions (e.g., production, immune recruitment, and contact, as labeled), dashed arrows denote spatial migration (e.g., chemotactic migration), and arrows with an orthogonal line at the head indicate suppression (e.g., local killing). This feedback structure is represented mathematically by the reaction–diffusion–chemotaxis system (1).

The biological feedback structure underlying the model is summarized in Figure 1. The model is a computational oncology framework coupling tumour growth, immune killing, chemokine production, immune recruitment, and chemotactic immune migration.

The biological interpretation of the model is as follows. Tumour cells proliferate locally and are limited by tissue-level carrying capacity, so the baseline tumour dynamics are represented by logistic growth. Immune effector cells kill tumour cells through local tumour–immune contact, producing a negative interaction term in the tumour equation. Chemokines recruit immune cells into the tumour microenvironment and create spatial gradients that direct immune-cell migration. Thus, the immune population is influenced both by local source terms, representing recruitment or activation, and by chemotactic drift along the chemokine gradient. Finally, chemokines are produced by tumour cells and by tumour–immune interactions, while natural degradation or clearance reduces the chemokine concentration.

In schematic form, the biological mechanisms are

Tgrowslogistically,EkillsT,ArecruitsandattractsE,TandETproduceA.

These assumptions lead naturally to a reaction–diffusion–chemotaxis system: diffusion accounts for undirected spatial spreading, reaction terms describe local tumour–immune–chemokine interactions, and chemotaxis represents directed immune migration toward increasing chemokine concentration. The purpose of the paper is to analyze this model mathematically, identify thresholds separating homogeneous and spatially heterogeneous regimes, and construct a numerical method that respects the biological structure of the equations.

### 1.3. Related Work and Modelling Context

The present work sits at the intersection of several active research areas, which we briefly survey here to situate our contribution.

Mathematical and computational oncology.

Quantitative modelling has become central to the study of cancer as a multi-scale, spatially structured process. Integrative and computational frameworks combine mechanistic models with experimental and clinical data to interrogate tumour initiation, progression, heterogeneity, and treatment response [18,19,20,21,22]. The recent literature emphasizes both the maturation of mechanistic models toward clinical decision support and the increasing integration of mechanistic and data-driven approaches, including the community roadmap of Rockne et al. [23] and the emerging program of mechanistic learning [24,25,26,27]. The model studied here is mechanistic and analytically tractable, in the spirit of this broader effort to extract interpretable thresholds and mechanisms from minimal but biologically grounded systems.

Tumour–immune interaction models.

Dynamical models of tumour–immune competition originate with the work of Kuznetsov et al. [28], whose ordinary-differential-equation model of the cytotoxic T-lymphocyte response reproduces dormancy, immune escape, and recurrent dynamics. Subsequent models incorporated immunotherapy and immune recruitment [29,30,31,32,33]; a broad survey of non-spatial tumour–immune models is given by Eftimie et al. [34]. These models capture the temporal balance between tumour growth and immune control that our homogeneous equilibrium analysis also exhibits, but they do not resolve spatial organization.

Spatial tumour modelling and the microenvironment.

Spatially resolved models describe how local cell–signal interactions generate tissue-scale structure. Reaction–diffusion and taxis-based models of tumour-induced angiogenesis [35,36,37,38], avascular tumour growth [39,40,41,42], and the spatio-temporal response of cytotoxic T-lymphocytes to a solid tumour [11] demonstrate that directed migration and diffusible signals can produce heterogeneous invasion and immune infiltration patterns, including travelling waves and irregular spatial distributions. Agent-based and hybrid platforms such as PhysiCell [43,44] complement continuum approaches for the multicellular tumour microenvironment. Furthermore, recent coupled PDE–agent-based frameworks have rigorously elucidated the bidirectional feedback between hypoxia-driven angiogenesis and therapeutic resistance, demonstrating how spatial vascular heterogeneity and resistant niches emerge from multi-scale continuous and stochastic interactions [45]. Our model contributes a continuum treatment in which the spatial structure arises specifically from chemokine-directed immune chemotaxis.

Chemokines, immune infiltration, and hot-versus-cold tumours.

On the biological side, chemokines are the principal guidance cues that recruit and position immune effector cells within the tumour microenvironment [46,47,48,49]. The density, location, and organization of tumour-infiltrating lymphocytes are strongly prognostic [50,51,52,53], motivating the now-standard classification of tumours as immune “hot”, “altered”, or “cold” according to their infiltration state [54]. A central obstacle to immunotherapy is immune exclusion, in which effector T cells are present but physically kept away from cancer cells by features of the microenvironment [55,56,57,58]. The transition between spatially homogeneous coexistence and chemotaxis-driven heterogeneity studied in this paper is a mathematical caricature of precisely this hot/cold, infiltrated/excluded dichotomy.

Chemotaxis equations and their numerical analysis.

Mathematically, the immune chemotaxis term places the model within the Keller–Segel family of chemotaxis systems [59,60,61,62], for which aggregation, blow-up, and boundedness under logistic damping or critical sensitivity have been extensively analyzed [63,64,65,66]. Because chemotaxis systems concentrate mass and can form steep gradients, specialized structure-preserving discretizations are required: conservative finite-volume schemes [67], positivity preserving central-upwind schemes [68], and conservative upwind finite-element methods [69] have all been developed for Keller–Segel-type models. Our finite-volume scheme with upwind chemotactic flux and discrete positivity and residual diagnostics follows this line.

Contribution relative to the Special Issue.

This paper contributes to advanced computational oncology in three ways. First, it formulates a minimal tumour–immune–chemokine reaction–diffusion–chemotaxis model that couples immune-mediated tumour killing, chemokine-mediated immune recruitment, and directed immune migration along chemokine gradients. Second, it derives analytically interpretable thresholds for tumour-free control, homogeneous tumour–immune coexistence, and chemotaxis-driven finite-wavelength instability, thereby connecting biological feedback mechanisms with stability and pattern-formation criteria. Third, it implements a conservative finite-volume scheme with no-flux boundary treatment, upwind chemotactic flux, positivity monitoring, mass-balance diagnostics, and post hoc residual certification. These components link mathematical modelling, PDE-based stability analysis, and numerically reliable simulation in a form that is directly aligned with computational oncology studies of tumour-related reaction–diffusion and chemotaxis systems.

### 1.4. Outline

Section 2 formulates the dimensional model, states the boundary and initial conditions, and non-dimensionalizes the system. Section 3 presents the structure-preserving finite-volume scheme. Section 4 establishes local solvability, positivity, and a priori estimates; the technical proofs are collected in Appendix A. Section 5 identifies the equilibria and the tumour-free immune-control threshold. Section 6 derives the dispersion relation about coexistence, with the full Routh–Hurwitz algebra given in Appendix B. Section 7 reports the numerical experiments, each verifying a specific theoretical result. Section 8 concludes.

## 2. Methods (Model Formulation)

### 2.1. Dimensional Tumour–Immune–Chemokine Model

Let $\Omega \subset R^{d}$, d∈{1,2,3}, be a bounded smooth tissue domain with outward unit normal vector *n* on ∂Ω. We denote by T(t,x), E(t,x), and A(t,x) the tumour-cell density, immune effector-cell density, and chemokine concentration, respectively, at time t≥0 and position x∈Ω.

We model tumour–immune–chemokine interactions by the following reaction–diffusion–chemotaxis system:

(1) $$\begin{array}{cc} T_{t} & =d_{T}\Delta T+rT\left(1-\frac{T}{K}\right)-\kappa ET,\\ E_{t} & =d_{E}\Delta E-\chi \nabla \cdot \left(E\nabla A\right)+s_{0}+s_{1}\frac{A}{\eta +A}-\mu E-\rho ET,\\ A_{t} & =d_{A}\Delta A+\alpha_{T}T+\alpha_{C}ET-\lambda A. \end{array}$$

**Figure 2:**
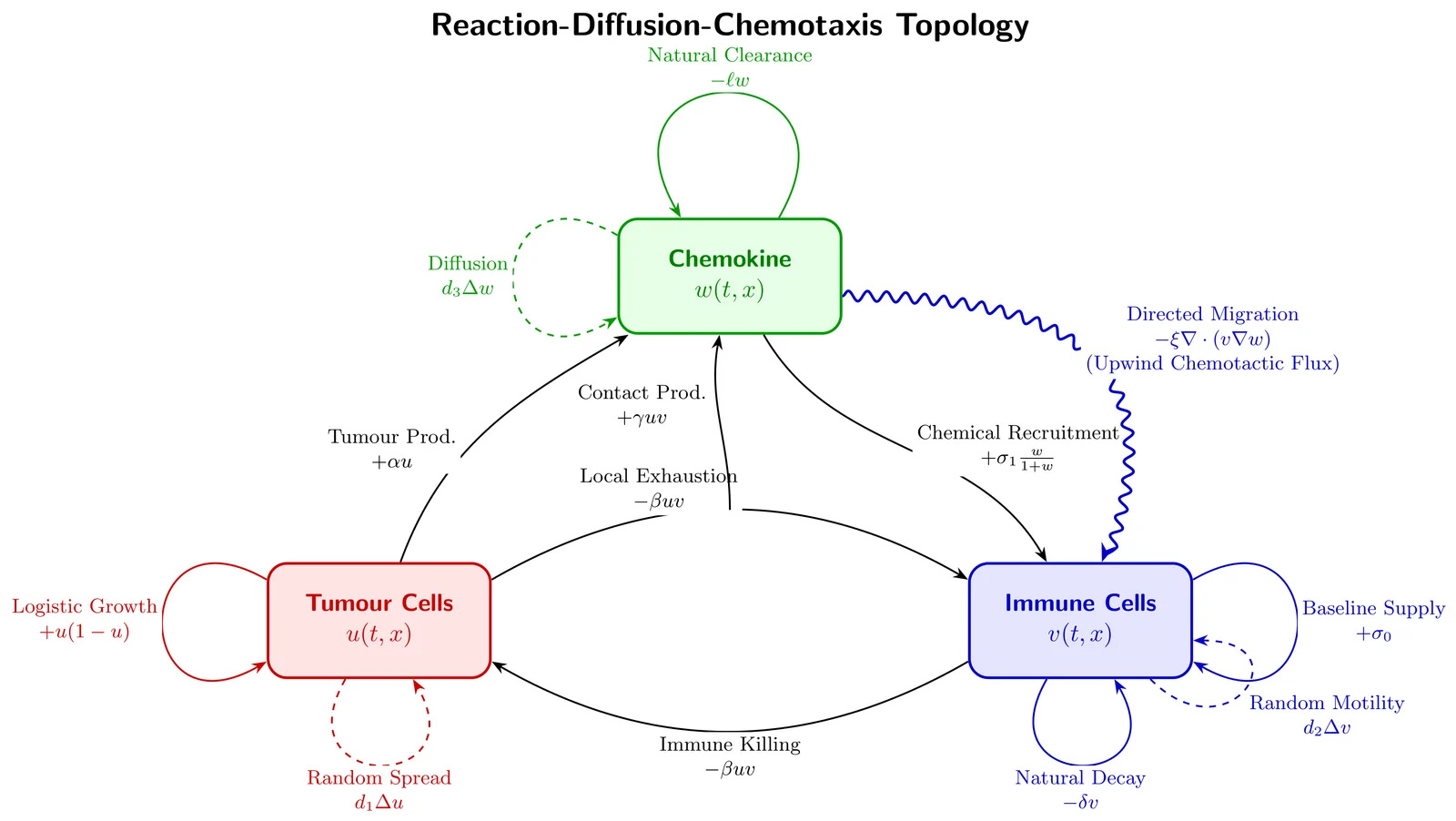
Schematic representation of the reaction–diffusion–chemotaxis topology for the tumour–immune–chemokine interactions. Colors denote the specific components and their intrinsic dynamics: red for tumour cells (*u*), blue for immune cells (*v*), and green for the chemokine (*w*). Black arrows indicate inter-population interactions, such as immune killing or chemical recruitment. Regarding the line styles, solid lines indicate local reaction kinetics, such as population growth, cell death, and chemical secretion. Dashed lines represent random spatial motility (diffusion), while the wavy blue line illustrates the directed chemotactic migration of immune cells up the chemokine gradient. The non-dimensional variables (u,v,w) depicted here correspond to the populations (T,E,A).

Here $d_{T},d_{E},d_{A}>0$ are diffusion coefficients. The tumour population grows logistically with intrinsic growth rate r>0 and carrying capacity K>0. The term −κET represents immune-mediated tumour killing, with the killing rate κ>0. The immune population diffuses with the coefficient $d_{E}>0$ and moves chemotactically in response to the chemokine gradient ∇A, with the chemotactic sensitivity χ≥0. The source term

$$s_{0}+s_{1}\frac{A}{\eta +A}$$

represents baseline immune influx together with chemokine-enhanced immune recruitment. The parameter $s_{0}\geq 0$ is the baseline immune supply, $s_{1}\geq 0$ is the maximal chemokine-induced recruitment rate, and η>0 is the half-saturation constant. Immune cells are removed at the rate μ>0 and may be locally exhausted or lost through tumour–immune interaction at the rate ρ>0. The chemokine is produced by tumour cells at the rate $\alpha_{T}>0$ and by tumour–immune interactions at rate $\alpha_{C}>0$, and is degraded or cleared at the rate λ>0. To visually summarize the complex spatial and biological interactions captured by the system (1), we present a schematic diagram of the reaction–diffusion–chemotaxis topology in Figure 2.

The model is intentionally minimal. It retains the mechanisms needed to describe spatial tumour growth, immune killing, chemokine-mediated immune recruitment, and directed immune migration, while remaining analytically tractable and numerically testable. In particular, the chemotaxis term −χ∇·(E∇A) is the mechanism through which chemokine gradients can amplify spatial heterogeneity and generate immune infiltration or immune-exclusion patterns.

### 2.2. Boundary and Initial Conditions

We impose biologically natural no-flux boundary conditions. These conditions describe a closed tissue region across whose boundary there is no net movement of tumour cells, immune cells, or chemokines:

(2) $$\nabla T\cdot n=0,\;(d_{E}\nabla E-\chi E\nabla A)\cdot n=0,\;\nabla A\cdot n=0\;\mathrm{on}\;\partial \Omega ,\;t>0.$$

The immune-cell condition is written in conservative form because the total immune flux is

$$J_{E}=-d_{E}\nabla E+\chi E\nabla A.$$

Thus, $J_{E}\cdot n=0$ is equivalent to

$$(d_{E}\nabla E-\chi E\nabla A)\cdot n=0.$$

This is the physical no-flux condition for the chemotactic immune population.

The initial data are assumed to satisfy

(3) $$T\left(0,x\right)=T_{0}\left(x\right),\;E\left(0,x\right)=E_{0}\left(x\right),\;A\left(0,x\right)=A_{0}\left(x\right),\;x\in \Omega ,$$

where

$$T_{0}\left(x\right)\geq 0,\;E_{0}\left(x\right)\geq 0,\;A_{0}\left(x\right)\geq 0\;\mathrm{for}\;\mathrm{all}\;x\in \Omega .$$

When classical solutions are considered, the initial data are also assumed to be sufficiently smooth and compatible with the boundary conditions.

For the eigenfunction-based linear stability calculation in Section 6, we impose the stronger componentwise Neumann condition

∇T·n=∇E·n=∇A·n=0on∂Ω.

This condition implies the natural no-flux condition (2) whenever ∇A·n=0. Its purpose is analytical: it allows the Neumann Laplacian eigenbasis to be used directly in the linear stability and dispersion-relation calculations. Table 1 lists dimensional parameters in the tumour–immune–chemokine model.

### 2.3. Non-Dimensionalization

We now non-dimensionalize (1). Let

$$\tilde{t}=rt,\;\tilde{x}=\frac{x}{L},\;u=\frac{T}{K},\;v=\frac{\kappa }{r}E,\;w=\frac{A}{\eta },$$

where L>0 is a reference tissue length scale. In these variables, *u* is the tumour density normalized by the carrying capacity, *v* is the immune density scaled by the tumour-killing rate relative to tumour growth, and *w* is the chemokine concentration normalized by the immune-recruitment half-saturation constant.

Dropping tildes after re-scaling, we obtain

(4) $$\begin{array}{cc} u_{t} & =d_{1}\Delta u+u\left(1-u-v\right),\\ v_{t} & =d_{2}\Delta v-\xi \nabla \cdot \left(v\nabla w\right)+\sigma_{0}+\sigma_{1}\frac{w}{1+w}-\delta v-\beta uv,\\ w_{t} & =d_{3}\Delta w+\alpha u+\gamma uv-lw. \end{array}$$

The non-dimensional diffusion coefficients are

$$d_{1}=\frac{d_{T}}{rL^{2}},\;d_{2}=\frac{d_{E}}{rL^{2}},\;d_{3}=\frac{d_{A}}{rL^{2}}.$$

The non-dimensional chemotactic sensitivity is

$$\xi =\frac{\chi \eta }{rL^{2}}.$$

The immune recruitment, decay, and tumour–immune loss parameters are

$$\sigma_{0}=\frac{\kappa s_{0}}{r^{2}},\;\sigma_{1}=\frac{\kappa s_{1}}{r^{2}},\;\delta =\frac{\mu }{r},\;\beta =\frac{\rho K}{r}.$$

The chemokine production and degradation parameters are

$$\alpha =\frac{\alpha_{T}K}{r\eta },\;\gamma =\frac{\alpha_{C}K}{\kappa \eta },\;l=\frac{\lambda }{r}.$$

The non-dimensional no-flux boundary conditions become

(5) $$\nabla u\cdot n=0,\;(d_{2}\nabla v-\xi v\nabla w)\cdot n=0,\;\nabla w\cdot n=0\;\mathrm{on}\;\partial \Omega ,\;t>0.$$

For the analytical linear stability calculation, we again use the stronger componentwise Neumann condition

(6) ∇u·n=∇v·n=∇w·n=0on∂Ω.

Table 2 lists non-dimensional parameters. The non-dimensional system (4) is the main model analyzed in the remainder of the paper. The first equation describes logistic tumour growth and immune-mediated tumour removal. The second equation describes immune-cell diffusion, chemotactic migration, chemokine-mediated recruitment, natural immune-cell loss, and tumour-induced immune loss. The third equation describes chemokine diffusion, production by tumour cells and tumour–immune interactions, and chemokine degradation. This formulation makes explicit the mechanisms by which chemokine-mediated immune recruitment and chemotactic migration may stabilize a homogeneous tumour–immune coexistence state or destabilize it toward spatially heterogeneous patterns.

Biological plausibility of the non-dimensional base set.

The non-dimensional groups inherit clear biological orderings. Because chemokine molecules diffuse far faster than immune cells migrate, which in turn move far faster than bulk tumour cells, one has $d_{A}\gg d_{E}\gg d_{T}$ and hence $d_{3}\gg d_{2}\gg d_{1}$; the base values $d_{3}=1$, $d_{2}=0.1$, and $d_{1}=0.01$ reproduce exactly this three-order separation. A representative dimensional set ($r=0.2\;{\mathrm{day}}^{-1}$, tissue scale L≈6–7mm, motilities $d_{T}:d_{E}:d_{A}\approx 0.1:1:10\;\mu m^{2}\;s^{-1}$, immune loss $\mu =0.06\;{\mathrm{day}}^{-1}$) maps onto the base values $d_{1}=0.01$, $d_{2}=0.10$, $d_{3}=1.00$, δ=0.30, and l=1.00. We note one deliberate simplification: fast chemokine turnover ($\lambda ∼1\;h^{-1}$) would give l≫1, placing the chemokine near a quasi-steady state; we retain l=O(1) for analytical transparency, which re-scales but does not remove the chemotaxis-driven finite-wavelength instability.

## 3. Methods (Numerical Scheme)

This section describes the numerical method used to simulate the non-dimensional tumour–immune–chemokine system (4). The numerical method is designed around three requirements: (i) $u_{h}$, $v_{h}$, $w_{h}\geq 0$, (ii) discrete no-flux boundary conditions, (iii) mass-balance and residual diagnostics. These requirements are important because negative cell densities or artificial boundary fluxes would have no biological interpretation and could distort the onset of spatial tumour–immune patterns. Chemotaxis systems are well known to concentrate mass and form steep gradients, which has motivated a substantial literature on structure-preserving discretizations for Keller–Segel-type models [67,68,69].

For clarity, we first describe the method on a one-dimensional interval Ω=(0,L). The extension to rectangular two-dimensional domains is obtained by applying the same finite-volume construction in each coordinate direction.

### 3.1. Finite-Volume Discretization and No-Flux Treatment

Let N∈N, h=L/N, and define the cell centres

$$x_{i}=\left(i-\frac{1}{2}\right)h,\;i=1,\ldots ,N.$$

Let $u_{i}\left(t\right)$, $v_{i}\left(t\right)$, and $w_{i}\left(t\right)$ denote cell averages over the finite volume

$$C_{i}=\left(x_{i-1/2},x_{i+1/2}\right).$$

The semi-discrete finite-volume scheme is written in conservative form as

(7) $$\frac{du_{i}}{dt}=-\frac{F_{i+1/2}^{u}-F_{i-1/2}^{u}}{h}+u_{i}\left(1-u_{i}-v_{i}\right),$$

(8) $$\frac{dv_{i}}{dt}=-\frac{F_{i+1/2}^{v}-F_{i-1/2}^{v}}{h}+\sigma_{0}+\sigma_{1}\frac{w_{i}}{1+w_{i}}-\delta v_{i}-\beta u_{i}v_{i},$$

and

(9) $$\frac{dw_{i}}{dt}=-\frac{F_{i+1/2}^{w}-F_{i-1/2}^{w}}{h}+\alpha u_{i}+\gamma u_{i}v_{i}-lw_{i}.$$

The tumour and chemokine diffusive fluxes are approximated by centered finite differences:

(10) $$F_{i+1/2}^{u}=-d_{1}\frac{u_{i+1}-u_{i}}{h},\;F_{i+1/2}^{w}=-d_{3}\frac{w_{i+1}-w_{i}}{h}.$$

The immune-cell flux contains both diffusion and chemotaxis. We write the continuous immune flux as

$$J_{v}=-d_{2}\nabla v+\xi v\nabla w.$$

Accordingly, the numerical immune flux is

(11) $$F_{i+1/2}^{v}=-d_{2}\frac{v_{i+1}-v_{i}}{h}+\xi v_{i+1/2}^{\mathrm{up}}\frac{w_{i+1}-w_{i}}{h}.$$

Here $v_{i+1/2}^{\mathrm{up}}$ is the upwind value selected according to the discrete chemotactic velocity

$$a_{i+1/2}=\xi \frac{w_{i+1}-w_{i}}{h}.$$

Specifically,

(12) $$v_{i+1/2}^{\mathrm{up}}=\left\{\begin{array}{cc} v_{i}, & a_{i+1/2}\geq 0,\\ v_{i+1}, & a_{i+1/2}<0. \end{array}\right.$$

The chemotactic flux is therefore discretized by an upwind finite-volume flux. This choice is first-order in the advective chemotaxis term but improves robustness and positivity in parameter regimes where steep immune gradients form.

The no-flux boundary conditions are imposed by setting the boundary fluxes to zero:

(13) $$F_{1/2}^{u}=F_{N+1/2}^{u}=0,\;F_{1/2}^{v}=F_{N+1/2}^{v}=0,\;F_{1/2}^{w}=F_{N+1/2}^{w}=0.$$

**Figure 3:**
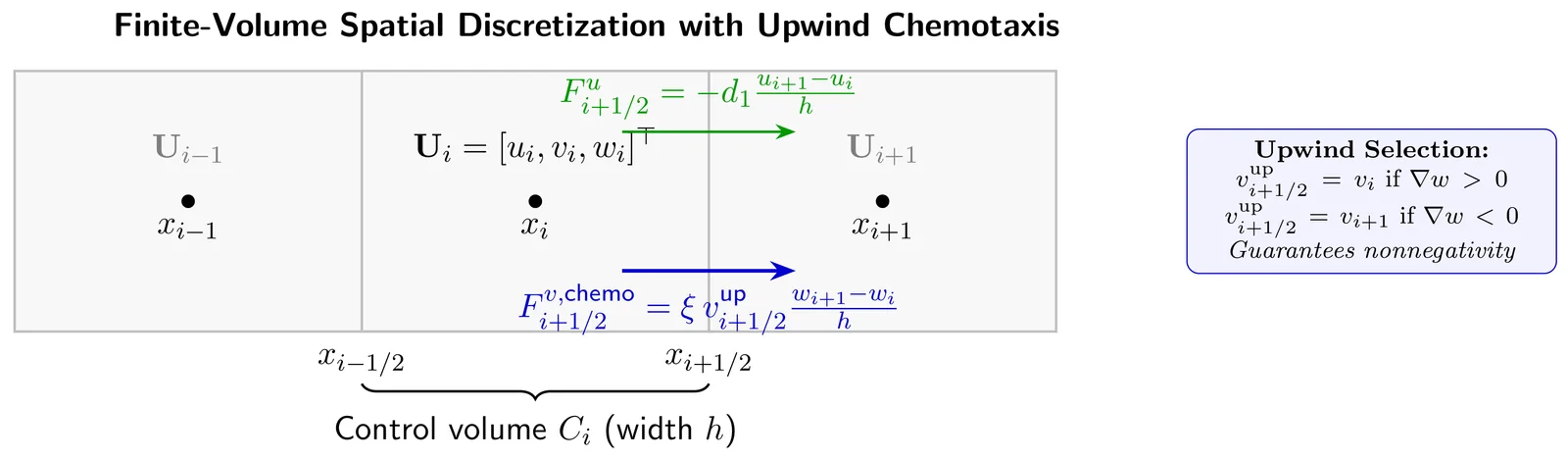
Schematic of the 1D finite-volume spatial discretization framework. The domain is divided into control volumes $C_{i}$ of uniform width *h*, with nodes located at the cell centers $x_{i}$ and boundaries at the interfaces $x_{i\pm 1/2}$. A centered difference approximation evaluates the diffusion flux $F_{i+1/2}^{u}$. In contrast, an upwind scheme dictates the chemotactic flux $F_{i+1/2}^{v,\mathrm{chemo}}$ to effectively capture directed migration and ensure the strict nonnegativity of cell densities.

This implementation is conservative at the discrete level. Summing (7)–(9) over all cells gives cancellation of interior fluxes and leaves only reaction contributions. The finite-volume spatial discretization framework is illustrated in Figure 3.

### 3.2. Mass-Balance Diagnostics

The discrete masses are defined by

$$M_{u}\left(t\right)=h\sum_{i=1}^{N}u_{i}\left(t\right),\;M_{v}\left(t\right)=h\sum_{i=1}^{N}v_{i}\left(t\right),\;M_{w}\left(t\right)=h\sum_{i=1}^{N}w_{i}\left(t\right).$$

Because of the conservative finite-volume form and the no-flux boundary treatment, the numerical masses satisfy the discrete balance laws

(14) $$\frac{dM_{u}}{dt}=h\sum_{i=1}^{N}u_{i}\left(1-u_{i}-v_{i}\right),$$

(15) $$\frac{dM_{v}}{dt}=h\sum_{i=1}^{N}\left(\sigma_{0}+\sigma_{1}\frac{w_{i}}{1+w_{i}}-\delta v_{i}-\beta u_{i}v_{i}\right),$$

and

(16) $$\frac{dM_{w}}{dt}=h\sum_{i=1}^{N}\left(\alpha u_{i}+\gamma u_{i}v_{i}-lw_{i}\right).$$

These identities are used as mass-balance diagnostics in the numerical experiments. In particular, the discrepancy between the left-hand side and the right-hand side of (14)–(16) provides a simple check on temporal accuracy and boundary-flux implementation.

### 3.3. BDF Time Stepping with Positivity Diagnostics

The semi-discrete system (7)–(9) is stiff when diffusion is strong, the mesh is fine, or chemotactic gradients are large. Therefore, we use an implicit backward differentiation formula (BDF) time discretization. Let

$$U^{n}={\left(u_{1}^{n},\ldots ,u_{N}^{n},v_{1}^{n},\ldots ,v_{N}^{n},w_{1}^{n},\ldots ,w_{N}^{n}\right)}^{⊤}$$

denote the vector of all cell averages at time $t_{n}$. In compact form, the semi-discrete system is

$$\frac{dU}{dt}=R_{h}\left(U\right),$$

where $R_{h}$ contains the finite-volume fluxes and reaction terms.

For example, the first-order BDF scheme is backward Euler:

(17) $$\frac{U^{n+1}-U^{n}}{\Delta t}=R_{h}\left(U^{n+1}\right).$$

In computations requiring higher temporal accuracy, a second-order BDF method is used after one backward Euler starting step:

(18) $$\frac{3U^{n+1}-4U^{n}+U^{n-1}}{2\Delta t}=R_{h}\left(U^{n+1}\right).$$

The nonlinear algebraic system at each time step is solved by Newton iteration with tolerance $\varepsilon_{\mathrm{newton}}$. Time steps are reduced if Newton iteration fails to converge or if the positivity diagnostic described below is violated beyond the prescribed tolerance.

We do not claim that the BDF method by itself guarantees positivity. Instead, positivity is promoted by the conservative finite-volume form, the no-flux boundary treatment, and the upwind chemotactic flux, and is monitored explicitly during the computation. At each time step we compute

(19) $$m_{\min}^{n}=\min\left\{\min_{i}u_{i}^{n},\;\min_{i}v_{i}^{n},\;\min_{i}w_{i}^{n}\right\}.$$

The run is accepted only if

(20) $$m_{\min}^{n}\geq -\varepsilon_{\mathrm{pos}}$$

for all reported time steps, where $\varepsilon_{\mathrm{pos}}$ is a small solver tolerance. In all reported runs, the minimum of each component remains nonnegative up to solver tolerance.

### 3.4. Residual Diagnostics

To distinguish genuine pattern formation from numerical artifacts, we compute post hoc residuals for the three equations. Let $D_{t}$, $\Delta_{h}$, and $\nabla_{h}\cdot$ denote the discrete time derivative, Laplacian, and divergence operators induced by the finite-volume method. The residuals are defined by

(21) $$R_{u}^{n}=D_{t}u^{n}-d_{1}\Delta_{h}u^{n}-u^{n}\left(1-u^{n}-v^{n}\right),$$

(22) $$R_{v}^{n}=D_{t}v^{n}-d_{2}\Delta_{h}v^{n}+\xi \nabla_{h}\cdot \left(v^{n}\nabla_{h}w^{n}\right)-\sigma_{0}-\sigma_{1}\frac{w^{n}}{1+w^{n}}+\delta v^{n}+\beta u^{n}v^{n},$$

and

(23) $$R_{w}^{n}=D_{t}w^{n}-d_{3}\Delta_{h}w^{n}-\alpha u^{n}-\gamma u^{n}v^{n}+lw^{n}.$$

We report the normalized residual indicator

(24) $$E_{\mathrm{res}}^{n}=\frac{∥R_{u}^{n}{∥}_{2}+∥R_{v}^{n}{∥}_{2}+{∥R_{w}^{n}∥}_{2}}{1+∥u^{n}{∥}_{2}+∥v^{n}{∥}_{2}+{∥w^{n}∥}_{2}}.$$

Together with the positivity diagnostic and the mass-balance identities, $E_{\mathrm{res}}^{n}$ provides a numerical certificate that the computed spatial patterns are consistent with the PDE system and not merely artifacts of the discretization.

### 3.5. Consistency, Positivity, and Convergence Status of the Scheme

We complement the a posteriori diagnostics with an a priori analysis of the finite-volume discretization.

Consistency. On a smooth solution, Taylor expansion of the fluxes about each face gives, for the diffusion and reaction terms, a local truncation error of order $O(h^{2})$ (centred face gradients and midpoint reaction quadrature are second order). The first-order upwind chemotactic flux (11) and (12) contributes a numerical-diffusion error of size $\frac{h}{2}\;|a_{i+1/2}|\;\partial_{xx}v\left(x_{i}\right)+\frac{h}{2}\partial_{x}\left(|a\right|\left(x_{i}\right))\partial_{x}v\left(x_{i-1/2}\right)+O\left(h^{2}\right)$, i.e., O(h) where the chemotactic drift $a=\xi \partial_{x}w$ is nonzero. Hence the semi-discrete scheme is consistent, with local truncation error
  $$\begin{array}{cc} \tau_{h} & =O(h)\;\mathrm{in}\;\mathrm{general},\\ \tau_{h} & =O(h^{2})\;\mathrm{where}\;\mathrm{the}\;\mathrm{leading}\;\mathrm{upwind}\;\mathrm{defect}\;\partial_{x}\left(\left|a\right|\partial_{x}v\right)\;\mathrm{vanishes}. \end{array}$$
  This is consistent with the observed order 1.92 in Section 7.6, where the smooth diffusion–reaction part dominates; the order degrades toward 1 only in strongly chemotaxis-dominated regimes with steep immune gradients.
  Positivity with a precise step-size condition. We prove that the explicit scheme preserves nonnegativity under a computable step-size restriction. Consider the fully explicit forward-Euler update of the finite-volume scheme on a uniform Cartesian mesh of spacing *h* in dimension D∈{1,2,3}, with no-flux boundaries imposed as zero boundary-face fluxes. For an interior face in coordinate direction *e* let $a_{i+\frac{1}{2}e}=\xi \;\left(w_{i+e}-w_{i}\right)/h$ be the discrete chemotactic velocity, and write $a^{\pm }=\max\left\{\pm a,0\right\}$. Let $n_{i}\leq 2D$ be the number of present neighbours of cell *i*, and set
  $$M_{u}^{n}=\max_{i}u_{i}^{n},\;M_{v}^{n}=\max_{i}v_{i}^{n},\;G^{n}=\max_{\mathrm{faces}}\frac{|w_{j}^{n}-w_{i}^{n}|}{h}.$$

**Proposition** **1** (discrete positivity)**.** *Assume $u^{n},v^{n},w^{n}\geq 0$ componentwise. If*

(25) $$\Delta t\left(\frac{2D\max\{d_{1},d_{2},d_{3}\}}{h^{2}}+\frac{2D\;\xi \;G^{n}}{h}+\max\left\{\;M_{u}^{n}+M_{v}^{n},\;\delta +\beta M_{u}^{n},\;l\;\right\}\right)\leq 1,$$

*then $u^{n+1},v^{n+1},w^{n+1}\geq 0$ componentwise. Hence if* (25) *holds at every step the scheme preserves nonnegativity for all n.*

**Proof.**  The upwind rule (12) writes the discrete chemotactic flux as $a_{i+\frac{1}{2}e}\;v_{i+\frac{1}{2}e}^{\mathrm{up}}=a_{i+\frac{1}{2}e}^{+}v_{i}-a_{i+\frac{1}{2}e}^{-}v_{i+e}$. Substituting into the update and collecting terms, each component takes the form

$$c_{i}^{\;n+1}=\sum_{j∼i}\theta_{ij}\;c_{j}^{\;n}+\Delta t\;S_{i}+\left(1-\Delta t\;\kappa_{i}\right)c_{i}^{\;n},\;\theta_{ij}\geq 0,\;\;S_{i}\geq 0,$$

as follows.

*Tumour.* $u_{i}^{n+1}=\frac{\Delta t\;d_{1}}{h^{2}}\sum_{j∼i}u_{j}^{\;n}+\left(1-\Delta t\;\kappa_{i}^{u}\right)u_{i}^{\;n}$, with $\kappa_{i}^{u}=\frac{d_{1}n_{i}}{h^{2}}-\left(1-u_{i}^{n}-v_{i}^{n}\right)\leq \frac{2Dd_{1}}{h^{2}}+M_{u}^{n}+M_{v}^{n}$ (the growth $+u_{i}$ is retained inside the centre weight and only lowers $\kappa_{i}^{u}$).

*Chemokine.* $w_{i}^{n+1}=\frac{\Delta t\;d_{3}}{h^{2}}\sum_{j∼i}w_{j}^{\;n}+\Delta t\left(\alpha u_{i}^{n}+\gamma u_{i}^{n}v_{i}^{n}\right)+\left(1-\Delta t\;\kappa_{i}^{w}\right)w_{i}^{\;n}$, with $\kappa_{i}^{w}=\frac{d_{3}n_{i}}{h^{2}}+l\leq \frac{2Dd_{3}}{h^{2}}+l$ and source ≥0.

*Immune.* The neighbour weight of $v_{i-e}$ is $\Delta t(\frac{d_{2}}{h^{2}}+\frac{a_{i-\frac{1}{2}e}^{+}}{h})\geq 0$ and of $v_{i+e}$ is $\Delta t(\frac{d_{2}}{h^{2}}+\frac{a_{i+\frac{1}{2}e}^{-}}{h})\geq 0$; the source $\Delta t\left(\sigma_{0}+\sigma_{1}\frac{w_{i}}{1+w_{i}}\right)\geq 0$; and

$$\kappa_{i}^{v}=\frac{d_{2}n_{i}}{h^{2}}+\frac{1}{h}\sum_{e}(a_{i+\frac{1}{2}e}^{+}+a_{i-\frac{1}{2}e}^{-})+\delta +\beta u_{i}^{n}\leq \frac{2Dd_{2}}{h^{2}}+\frac{2D\;\xi G^{n}}{h}+\delta +\beta M_{u}^{n},$$

where $\sum_{e}(a_{i+\frac{1}{2}e}^{+}+a_{i-\frac{1}{2}e}^{-})\leq \sum_{\mathrm{faces}\;\mathrm{at}\;i}\left|a\right|\leq 2D\;\xi G^{n}$.

In every case the neighbour weights and sources are nonnegative and the centre weight is $1-\Delta t\;\kappa_{i}$. The bracket in (25) dominates each of $\kappa_{i}^{u},\kappa_{i}^{v},\kappa_{i}^{w}$, so (25) gives $\Delta t\;\kappa_{i}\leq 1$ for every cell and component; hence every centre weight is nonnegative. Thus $c_{i}^{\;n+1}$ is a nonnegative combination of nonnegative quantities, so $c_{i}^{\;n+1}\geq 0$. Induction on *n* completes the proof.    □

Because *v* has no a priori $L^{\infty }$ bound (only the mass bound of Proposition 3), the peak $M_{v}^{n}$ may grow as immune cells aggregate, and (25) then automatically reduces Δt—consistent with Remark 1; the tumour factor is controlled by the discrete logistic bound $M_{u}^{n}\leq \max\{1,∥u^{0}{∥}_{\infty }\}$. In the implementation the step is selected adaptively to satisfy (25) with a safety factor below one, so positivity holds by the Proposition. The implicit BDF integrator used for the stiff one-dimensional runs carries no such guarantee and is instead monitored a posteriori via (19) and (20).

Convergence status. For sufficiently smooth solutions, the spatial finite-volume operator is consistent, with truncation error O(h) in general because of the upwind chemotactic flux and $O(h^{2})$ when its leading first-order contribution vanishes. Proposition 1 establishes nonnegativity of the forward-Euler discretization under (25), while the conservative flux form gives the discrete mass-balance identities. These properties alone, however, do not constitute a convergence proof for the nonlinear cross-diffusion system. Such a proof would additionally require mesh-uniform stability estimates and either a discrete compactness argument or a direct error estimate around the classical solution; see, for example, [67,75]. We therefore interpret the grid-refinement results of Section 7.6 as numerical evidence of self-convergence. The observed order 1.92 reflects the smooth, diffusion-dominated regime considered there; the formal asymptotic spatial order of the upwind scheme is first order in general.

### 3.6. Extension to Two-Dimensional Rectangular Domains

On a rectangular domain

$$\Omega =\left(0,L_{x}\right)\times \left(0,L_{y}\right),$$

we use a Cartesian finite-volume grid with cell averages

$$u_{i,j}\left(t\right),\;v_{i,j}\left(t\right),\;w_{i,j}\left(t\right).$$

Diffusive fluxes are computed on cell faces by centered differences, while the chemotactic immune flux is computed face-by-face using the upwind value of *v* determined by the corresponding discrete chemotactic velocity. For example, in the *x*-direction,

$$F_{i+1/2,j}^{v,x}=-d_{2}\frac{v_{i+1,j}-v_{i,j}}{h_{x}}+\xi v_{i+1/2,j}^{\mathrm{up}}\frac{w_{i+1,j}-w_{i,j}}{h_{x}},$$

and the analogous formula is used in the *y*-direction. No-flux boundary conditions are again imposed by setting all boundary face fluxes to zero.

This dimension-by-dimension finite-volume construction preserves the same conservative structure as the one-dimensional scheme. The positivity, mass-balance, and residual diagnostics are computed in the same way, with sums taken over all grid cells.

## 4. Results (Mathematical Well-Posedness)

In this section, we establish the basic analytical properties of the non-dimensional tumour–immune–chemokine system (4) posed in a bounded smooth domain $\Omega \subset R^{d}$ with nonnegative initial data. The purpose of this section is to identify the mathematical regime in which the model is biologically meaningful: solutions exist at least locally, remain nonnegative, and satisfy basic a priori estimates. These estimates also provide the analytical foundation for the equilibrium and linear stability analysis in the following sections. The technical proofs are collected in Appendix A.

Throughout the paper we assume

$$d_{1},d_{2},d_{3}>0,\;\xi ,\sigma_{0},\sigma_{1},\delta ,\beta ,\alpha ,\gamma ,l\geq 0,\;\delta >0,\;l>0.$$

Unless otherwise stated, solutions are considered under the natural no-flux boundary conditions (5). For the eigenfunction-based linear stability calculation, we shall use the stronger componentwise Neumann condition (6) as already explained in Section 2.2.

### 4.1. Classical Solvability and Positivity

We first record the local classical solvability and positivity property of (4). The system is quasilinear only through the chemotactic flux in the *v*-equation. Since the principal diffusion matrix is triangular with positive diagonal entries $d_{1},d_{2},d_{3}$, the system is normally parabolic in the standard sense. Local solvability therefore follows from classical quasilinear parabolic theory [76], while nonnegativity follows from the quasi-positivity of the reaction terms and the no-flux boundary conditions.

**Theorem** **1** (Local classical solvability and positivity)**.** *Let $\Omega \subset R^{d}$ be a bounded domain with smooth boundary and let θ∈(0,1). Suppose that*

$$\left(u_{0},v_{0},w_{0}\right)\in C^{2+\theta }{\left(\bar{\Omega }\right)}^{3},\;u_{0},v_{0},w_{0}\geq 0\;\mathrm{in}\;\bar{\Omega },$$

*and assume that the initial data are compatible with the boundary conditions. Then there exists a maximal time $T_{\max}\in \left(0,\infty \right]$ and a unique nonnegative classical solution,*

$$\left(u,v,w\right)\in C_{\mathrm{loc}}^{1+\theta /2,\;2+\theta }{\left(\left(0,T_{\max}\right)\times \bar{\Omega }\right)}^{3},$$

*of* (4) *and* (5) *satisfying*

$$u\left(0,\cdot \right)=u_{0},\;v\left(0,\cdot \right)=v_{0},\;w\left(0,\cdot \right)=w_{0}.$$

*Moreover, either $T_{\max}=\infty$, or*

$$\underset{t↑T_{\max}}{\lim\;\sup}\left({∥u\left(t\right)∥}_{L^{\infty }\left(\Omega \right)}+{∥v\left(t\right)∥}_{L^{\infty }\left(\Omega \right)}+{∥w\left(t\right)∥}_{L^{\infty }\left(\Omega \right)}\right)=\infty .$$

The proof, based on normal parabolicity and the quasi-positivity of the reaction vector field, is given in Appendix A.1.

### 4.2. A Priori Estimates

We next state the estimates that are used throughout the rest of the paper. The first estimate is a uniform pointwise bound on the tumour density; its proof is given in Appendix A.2.

**Proposition** **2** (Tumour-density bound)**.** *Let (u,v,w) be a nonnegative classical solution of* (4) *on $\left[0,T_{\max}\right)$. Then*

$$0\leq u\left(t,x\right)\leq M_{u}\;\mathrm{for}\;\mathrm{all}\;\left(t,x\right)\in \left[0,T_{\max}\right)\times \bar{\Omega },$$

*where $M_{u}:=\max\{1,∥u_{0}{∥}_{L^{\infty }\left(\Omega \right)}\;\}$.*

The next estimates give global-in-time integral control of the immune and chemokine variables as long as the classical solution exists; their proofs are given in Appendix A.3 and Appendix A.4.

**Proposition** **3** (Immune and chemokine mass estimates)**.** *Let (u,v,w) be a nonnegative classical solution of* (4) *on $\left[0,T_{\max}\right)$, and let $M_{u}:=\max\{1,∥u_{0}{∥}_{L^{\infty }\left(\Omega \right)}\}$. Then, for all $t<T_{\max}$,*

(26) $$\frac{d}{dt}\int_{\Omega }v\left(t,x\right)\;dx\leq \left|\Omega \right|\left(\sigma_{0}+\sigma_{1}\right)-\delta \int_{\Omega }v\left(t,x\right)\;dx,$$

*and*

(27) $$\frac{d}{dt}\int_{\Omega }w\left(t,x\right)\;dx\leq \alpha M_{u}\left|\Omega \right|+\gamma M_{u}\int_{\Omega }v\left(t,x\right)\;dx-l\int_{\Omega }w\left(t,x\right)\;dx.$$

*Consequently,*

(28) $$\int_{\Omega }v\left(t,x\right)\;dx\leq e^{-\delta t}\int_{\Omega }v_{0}\left(x\right)\;dx+\frac{\left|\Omega |(\sigma_{0}+\sigma_{1}\right)}{\delta }\left(1-e^{-\delta t}\right),$$

*and there exists a constant $C_{w}>0$, depending only on the parameters, Ω, and the initial masses, such that*

(29) $$\underset{0<t<T_{\max}}{\sup}\int_{\Omega }w\left(t,x\right)\;dx\leq C_{w}.$$

**Remark** **1** (What the estimates do and do not prove)**.** *The estimates above provide tumour-density boundedness and global mass control for the immune and chemokine variables. They do not by themselves imply full global $L^{\infty }$-boundedness of the three-component chemotaxis system in arbitrary spatial dimension. Full boundedness would require additional assumptions, such as stronger damping, small chemotactic sensitivity, entropy-type estimates, signal-dependent desensitization, or dimension-dependent regularity arguments [63,64]; see Appendix A.5 for a precise discussion. Accordingly, the results in the rest of the paper are stated for classical solutions on their interval of existence, or for globally defined bounded solutions when such boundedness is assumed or verified numerically.*

## 5. Results (Equilibria)

In this section, we identify the spatially homogeneous equilibria of the non-dimensional tumour–immune–chemokine system (4). Spatially homogeneous equilibria play two roles. Mathematically, they provide the base states for the linear stability and dispersion-relation analysis. Biologically, they correspond to different tumour–immune regimes: tumour-free control, spatially homogeneous coexistence, or possible transition toward spatially heterogeneous tumour–immune organization.

Throughout this section, a spatially homogeneous equilibrium is a constant triple

$$\left(u*,v*,w*\right)\in {\left[0,\infty \right)}^{3}$$

satisfying

(30) $$\left\{\begin{array}{c} 0=u*\left(1-u*-v*\right),\\ 0=\sigma_{0}+\sigma_{1}\frac{w*}{1+w*}-\delta v*-\beta u*v*,\\ 0=\alpha u*+\gamma u*v*-lw*. \end{array}\right.$$

### 5.1. Tumour-Free Equilibrium

The tumour-free equilibrium is obtained by setting u*=0. Since the chemokine equation then gives w*=0, the immune equation gives $v*=\frac{\sigma_{0}}{\delta }$. Thus,

(31) $$E_{0}=\left(0,\frac{\sigma_{0}}{\delta },0\right).$$

This equilibrium represents a tumour-free tissue state maintained by the baseline immune supply $\sigma_{0}$ and immune loss rate δ.

The stability of $E_{0}$ has a particularly simple interpretation. Near $E_{0}$, the tumour equation linearizes as

$$u_{t}=d_{1}\Delta u+\left(1-\frac{\sigma_{0}}{\delta }\right)u.$$

Therefore, tumour perturbations decay when the baseline immune level $\sigma_{0}/\delta$ exceeds the non-dimensional tumour growth strength 1, and they grow when it is below this level.

**Theorem** **2** (Tumour-free threshold)**.** *The tumour-free equilibrium $E_{0}$ is linearly stable if $\sigma_{0}>\delta$, linearly unstable if $\sigma_{0}<\delta$, and non-hyperbolic at $\sigma_{0}=\delta$. At this equilibrium, chemotaxis does not affect the linear tumour-invasion threshold.*

**Proof.**  Let

$$u=0+\tilde{u},\;v=\frac{\sigma_{0}}{\delta }+\tilde{v},\;w=0+\tilde{w}.$$

The linearization of (4) at $E_{0}$ is

(32) $$\left\{\begin{array}{c} {\tilde{u}}_{t}=d_{1}\Delta \tilde{u}+\left(1-\frac{\sigma_{0}}{\delta }\right)\tilde{u},\\ {\tilde{v}}_{t}=d_{2}\Delta \tilde{v}-\delta \tilde{v}+\sigma_{1}\tilde{w}-\xi \frac{\sigma_{0}}{\delta }\Delta \tilde{w}-\beta \frac{\sigma_{0}}{\delta }\tilde{u},\\ {\tilde{w}}_{t}=d_{3}\Delta \tilde{w}+\left(\alpha +\gamma \frac{\sigma_{0}}{\delta }\right)\tilde{u}-l\tilde{w}. \end{array}\right.$$

Using the Neumann Laplacian eigenbasis

$$-\Delta \phi_{k}=\mu_{k}\phi_{k},\;\partial_{n}\phi_{k}=0,\;0=\mu_{0}<\mu_{1}\leq \mu_{2}\leq \ldots ,$$

the tumour component has growth rate $\lambda_{k}^{\left(u\right)}=1-\frac{\sigma_{0}}{\delta }-d_{1}\mu_{k}$. The largest of these rates occurs at k=0 and equals $\lambda_{0}^{\left(u\right)}=1-\frac{\sigma_{0}}{\delta }$. Hence all tumour perturbations decay if $\sigma_{0}>\delta$, while the homogeneous tumour mode grows if $\sigma_{0}<\delta$.

The remaining two components form a triangularly forced stable subsystem once $\tilde{u}$ is fixed. Indeed, the diagonal decay rates for $\tilde{v}$ and $\tilde{w}$ contain $-\delta -d_{2}\mu_{k}$ and $-l-d_{3}\mu_{k}$, which are strictly negative. Therefore, the sign of the dominant eigenvalue is determined by $1-\sigma_{0}/\delta$. This proves the stated stability threshold.

Finally, the chemotactic contribution appears only in the immune equation through the linear term $-\xi \frac{\sigma_{0}}{\delta }\Delta \tilde{w}$. It does not enter the tumour growth rate $\lambda_{k}^{\left(u\right)}$. Thus chemotaxis does not affect the linear tumour-invasion threshold at $E_{0}$.    □

**Remark** **2** (Biological interpretation)**.** *The threshold $\sigma_{0}>\delta$ means that baseline immune supply is sufficiently large relative to immune loss to maintain a tumour-free state. Conversely, when $\sigma_{0}<\delta$, the tumour-free state is linearly unstable, and a small tumour perturbation can invade. Thus the ratio $\sigma_{0}/\delta$ acts as a non-dimensional immune-control threshold for tumour-free stability.*

*The tumour-free threshold $\sigma_{0}/\delta$ has a direct dimensional reading in tissue terms. Restoring units, $\sigma_{0}/\delta =\kappa s_{0}/\left(r\mu \right)=\kappa E_{0}/r$, where $E_{0}=s_{0}/\mu$ is the tumour-free immune density; thus $\sigma_{0}/\delta$ is the ratio of the baseline immune-mediated tumour kill rate $\kappa E_{0}$ to the tumour proliferation rate r. Control ($\sigma_{0}/\delta >1$) requires that resident immune surveillance remove tumour cells at least as fast as they divide. Using r∼0.1–$0.5\;{\mathrm{day}}^{-1}$ and effective immune-mediated kill rates $\kappa E_{0}∼0.05$–$0.6\;{\mathrm{day}}^{-1}$ from validated tumour–immune data [28,72], one finds $\sigma_{0}/\delta$ of order 0.1–10, i.e., straddling the control threshold. This near-threshold position is biologically meaningful: it is precisely why tumour fate is context-dependent, and why modest shifts in immune supply or turnover—as in the transition from immune “hot” to “cold” tumours [54]—can move a lesion across the surveillance boundary.*

### 5.2. Coexistence Equilibria

We next consider spatially homogeneous coexistence equilibria, for which

u*>0,v*>0,w*>0.

From the first equilibrium equation in (30), positivity of $u^{*}$ implies $1-u^{*}-v^{*}=0$, and therefore

(33) $$v^{*}=1-u^{*}.$$

Hence a positive coexistence equilibrium must satisfy $0<u^{*}<1$. The third equilibrium equation gives

(34) $$w^{*}=\frac{u^{*}\left[\alpha +\gamma \left(1-u^{*}\right)\right]}{l}.$$

Substituting (33) and (34) into the immune equilibrium equation yields a single scalar equation for $u^{*}$:

(35) F(u)=0,0<u<1,

where

(36) $$F\left(u\right)=\sigma_{0}+\sigma_{1}\frac{w(u)}{1+w(u)}-\left(\delta +\beta u\right)\left(1-u\right),$$

and

(37) $$w\left(u\right)=\frac{u[\alpha +\gamma (1-u)]}{l}.$$

**Proposition** **4** (Coexistence equilibria)**.** *Every spatially homogeneous coexistence equilibrium satisfies*

$$v*=1-u*,\;w*=\frac{u*\left[\alpha +\gamma \left(1-u*\right)\right]}{l},$$

where u*∈(0,1) is a zero of

$$F\left(u\right)=\sigma_{0}+\sigma_{1}\frac{w(u)}{1+w(u)}-\left(\delta +\beta u\right)\left(1-u\right),\;w\left(u\right)=\frac{u[\alpha +\gamma (1-u)]}{l}.$$

If $\sigma_{0}<\delta$ and F(1)>0, then at least one interior coexistence equilibrium $u^{*}\in \left(0,1\right)$ exists. The condition F(1)>0 holds whenever $\sigma_{0}>0$ or whenever $\sigma_{1}>0$ and α>0.

**Proof.**  Let (u*,v*,w*) be a coexistence equilibrium. Since u*>0, the first equation in (30) gives v*=1−u*. Since v*>0, it follows that u*∈(0,1). The third equation then gives

$$w*=\frac{\alpha u*+\gamma u*v*}{l}=\frac{u*\left[\alpha +\gamma \left(1-u*\right)\right]}{l}.$$

Substituting these expressions into the second equilibrium equation gives F(u*)=0, with *F* defined by (36).

For existence, observe that *F* is continuous on [0,1], with

$$w\left(0\right)=0,\;F\left(0\right)=\sigma_{0}-\delta <0,$$

under the hypothesis $\sigma_{0}<\delta$, while

$$F\left(1\right)=\sigma_{0}+\sigma_{1}\frac{w(1)}{1+w(1)},\;w\left(1\right)=\frac{\alpha }{l}.$$

If F(1)>0, the intermediate value theorem produces a zero u*∈(0,1). Finally, F(1)>0 holds whenever $\sigma_{0}>0$; and if $\sigma_{0}=0$ then $F\left(1\right)=\sigma_{1}\;\left(\alpha /l\right)/\left(1+\alpha /l\right)$, which is positive precisely when $\sigma_{1}>0$ and α>0.    □

**Remark** **3** (Boundary case)**.** If $\sigma_{0}=\sigma_{1}=0$, or if $\sigma_{1}>0$ but α=0 with $\sigma_{0}=0$, then F(1)=0 and the endpoint u=1 corresponds to an immune-free tumour-dominant boundary state (v*=0) rather than a positive coexistence state.

**Remark** **4** (Biological interpretation)**.** A coexistence equilibrium represents a spatially homogeneous balance between tumour growth, immune killing, immune recruitment, and chemokine signalling. The condition u*∈(0,1) means that the tumour persists below carrying capacity, while v*=1−u* means that immune pressure offsets the remaining tumour growth capacity. The scalar equation F(u)=0 balances immune recruitment, immune loss, and tumour-induced immune suppression. Thus coexistence is governed by the competition between immune supply and chemokine recruitment on one side, and immune decay and tumour-associated immune loss on the other.

## 6. Results (Linear Stability Analysis)

We now analyze the spatial stability of a homogeneous coexistence equilibrium (u*,v*,w*) of Proposition 4 with respect to finite-wavelength perturbations. This is the mechanism through which chemotaxis can convert a spatially homogeneous tumour–immune state into a spatially heterogeneous one. The algebraic details are collected in Appendix B; here we record the mode-wise stability matrix, the dispersion relation, and the resulting notion of a critical chemotactic sensitivity.

### 6.1. Mode-Wise Stability Matrix

**Lemma** **1** (The linearized flux condition reduces to Neumann)**.** Let (u*,v*,w*) be a homogeneous equilibrium and let $\left(\tilde{u},\tilde{v},\tilde{w}\right)$ be a perturbation satisfying the linearization of the physical no-flux conditions (2)*. Then the linearized conditions are exactly the componentwise Neumann conditions*

$$\nabla \tilde{u}\cdot n=\nabla \tilde{v}\cdot n=\nabla \tilde{w}\cdot n=0\;\mathrm{on}\;\partial \Omega .$$

**Proof.**  The tumour and chemokine conditions in (2) are already ∇u·n=0 and ∇w·n=0, which linearize to $\nabla \tilde{u}\cdot n=0$ and $\nabla \tilde{w}\cdot n=0$. The immune condition is $(d_{2}\nabla v-\xi v\nabla w)\cdot n=0$. Linearizing about $\left(v^{*},w^{*}\right)$ with $\nabla w^{*}=0$ gives

$$\left(d_{2}\nabla \tilde{v}-\xi v^{*}\nabla \tilde{w}-\xi \tilde{v}\;\nabla w^{*}\right)\cdot n=d_{2}\;\nabla \tilde{v}\cdot n-\xi v^{*}\;\nabla \tilde{w}\cdot n.$$

The second term vanishes because $\nabla \tilde{w}\cdot n=0$ from the chemokine condition, leaving $d_{2}\;\nabla \tilde{v}\cdot n=0$, i.e., $\nabla \tilde{v}\cdot n=0$ since $d_{2}>0$. Thus the coupled flux condition is equivalent, at the linear level about a homogeneous state, to the separate Neumann conditions used in the spectral analysis. In particular, the Neumann Laplacian eigenbasis $\left\{\phi_{k}\right\}$ diagonalizes all three linearized fluxes simultaneously, which is what makes the mode-by-mode reduction to $M_{k}$ in (38) legitimate.    □

Writing $u=u*+\tilde{u}$, $v=v*+\tilde{v}$, $w=w*+\tilde{w}$ and keeping first-order terms, the chemotactic flux linearizes as

$$-\xi \nabla \cdot \left(v\nabla w\right)\;⟼\;-\xi \nabla \cdot \left(v*\nabla \tilde{w}\right)=-\xi v*\Delta \tilde{w},$$

because ∇w*=0 at a homogeneous state. Projecting the linearized system onto the Neumann eigenbasis $-\Delta \phi_{k}=\mu_{k}\phi_{k}$, $\partial_{n}\phi_{k}=0$ (so that $\mu_{k}={\left(k\pi /L\right)}^{2}$ in one dimension and $\mu_{0}=0$), and using 1−2u*−v*=−u* at coexistence, the modal amplitudes $\left(U_{k},V_{k},W_{k}\right)$ satisfy $\frac{d}{dt}{\left(U_{k},V_{k},W_{k}\right)}^{⊤}=M_{k}\;{\left(U_{k},V_{k},W_{k}\right)}^{⊤}$ with

(38) $$M_{k}=\left(\begin{array}{ccc} -u^{*}-d_{1}\mu_{k} & -u* & 0\\ -\beta v* & -\left(\delta +\beta u*\right)-d_{2}\mu_{k} & \frac{\sigma_{1}}{{\left(1+w*\right)}^{2}}+\xi v*\mu_{k}\\ \alpha +\gamma v* & \gamma u* & -l-d_{3}\mu_{k} \end{array}\right).$$

The chemotactic sensitivity ξ enters $M_{k}$ only through the (2,3) entry, as the term $+\xi v*\mu_{k}$ is obtained from $-\xi v*\Delta \tilde{w}=\xi v*\mu_{k}\tilde{w}$. This contribution vanishes for the homogeneous mode $\mu_{0}=0$ and grows linearly with $\mu_{k}$; it is the sole route by which chemotaxis can destabilize finite spatial modes while leaving the spatially uniform mode unaffected.

### 6.2. Dispersion Relation and Critical Sensitivity

The equilibrium is linearly stable with respect to mode *k* if and only if all eigenvalues of $M_{k}$ lie in the open left half-plane. We define the dispersion relation

(39) ωk(ξ)=maxjReλj(Mk),

so that the coexistence state is linearly stable to all spatial perturbations precisely when $\omega_{k}\left(\xi \right)<0$ for every k≥0, and the critical chemotactic sensitivity is

(40) $$\xi_{c}=\inf\left\{\xi \geq 0:\;\max_{k\geq 1}\omega_{k}\left(\xi \right)=0\right\}.$$

The characteristic polynomial of $M_{k}$ is the monic cubic $\lambda^{3}+a_{1}\left(k\right)\lambda^{2}+a_{2}\left(k\right)\lambda +a_{3}\left(k\right)$, whose coefficients are computed in Appendix B. We have the following result:

**Proposition** **5** (Existence of a critical sensitivity and nature of the onset)**.** Assume the coexistence equilibrium $\left(u^{*},v^{*},w^{*}\right)$ is stable to the homogeneous mode, i.e., all eigenvalues of $M_{0}$ have negative real part, and adopt the notation (A11) *and* (A12)*. For each mode k≥1 the coefficients $a_{1}\left(k\right),a_{2}\left(k\right),a_{3}\left(k\right)$ of the characteristic cubic are smooth, and $a_{1}\left(k\right)>0$. Then:*

*(i)*  (Monotone destabilization.) Both $a_{2}\left(k;\xi \right)$ and $a_{3}\left(k;\xi \right)$ are affine and strictly decreasing in ξ, with slopes $\partial_{\xi }a_{2}\left(k\right)=-v^{*}\mu_{k}\;t<0$ and $\partial_{\xi }a_{3}\left(k\right)=-v^{*}\mu_{k}\;\left(A_{k}t-ps\right)$. Consequently the Hurwitz determinant $H_{k}\left(\xi \right):=a_{1}\left(k\right)a_{2}\left(k;\xi \right)-a_{3}\left(k;\xi \right)$ is affine in ξ and, for each fixed k with $\mu_{k}>0$, the map $\xi \mapsto \omega_{k}\left(\xi \right)$ is continuous and eventually positive.
*(ii)*  (Existence of $\xi_{c}$.) Since $\omega_{k}\left(0\right)<0$ for all k (homogeneous stability plus continuity of eigenvalues) and $\omega_{k}\left(\xi \right)\to +\infty$ as ξ→∞ for any k with $\mu_{k}>0$, the set $\left\{\xi \geq 0:\max_{k\geq 1}\omega_{k}\left(\xi \right)=0\right\}$ is nonempty and $\xi_{c}=\inf\left\{\xi :\max_{k\geq 1}\omega_{k}\left(\xi \right)\geq 0\right\}\in \left(0,\infty \right)$ is attained at some finite mode $k^{*}\geq 1$.
*(iii)*  (Stationary vs. oscillatory.) At $\xi =\xi_{c}$ the critical mode $k^{*}$ satisfies exactly one of:
  *(a)*  $a_{3}\left(k^{*};\xi_{c}\right)=0$, $a_{1}a_{2}-a_{3}>0$ (stationary/Turing: a simple real eigenvalue crosses 0);
  *(b)*  $H_{k^{*}}\left(\xi_{c}\right)=a_{1}a_{2}-a_{3}=0$, $a_{3}>0$ (oscillatory/Hopf: a complex pair crosses the imaginary axis at $\pm i\sqrt{a_{2}})$.
  The onset is stationary iff $a_{3}\left(k^{*};\xi_{c}\right)=0$ occurs at no larger ξ than $H_{k^{*}}=0$, and it is oscillatory otherwise. In case (b) the marginal frequency is $\omega^{*}=\sqrt{a_{2}\left(k^{*};\xi_{c}\right)}$.

**Proof.**  (i) From (A15) and (A16) and $r_{k}=\sigma_{1}/{\left(1+w^{*}\right)}^{2}+\xi v^{*}\mu_{k}$, only $r_{k}$ depends on ξ, linearly, so $a_{2}$ and $a_{3}$ are affine in ξ with the stated slopes; $\partial_{\xi }a_{2}=-t\;v^{*}\mu_{k}<0$ because $t=\gamma u^{*}>0$, and $\partial_{\xi }a_{3}=-v^{*}\mu_{k}\left(A_{k}t-ps\right)$ from (A21). Hence $H_{k}$ is affine in ξ. (ii) Homogeneous stability gives $\omega_{k}\left(0\right)<0$ for every *k* (the reaction Jacobian equals $M_{0}$ shifted by $-\mathrm{diag}\left(d_{i}\right)\mu_{k}≺0$, so adding diffusion cannot destabilize at ξ=0). As ξ→∞, the (2,3) entry $+\xi v^{*}\mu_{k}\to \infty$ forces an eigenvalue of $M_{k}$ into the right half-plane (e.g., $a_{3}\to -\infty$ when $A_{k}t-ps>0$, or $H_{k}\to -\infty$ when $\partial_{\xi }H_{k}<0$), so $\omega_{k}\left(\xi \right)\to +\infty$. Continuity of eigenvalues and compactness of the neutral set over the finitely many modes with $\mu_{k}$ below the eventual cutoff give a finite minimizing $k^{*}$ and $\xi_{c}\in \left(0,\infty \right)$. (iii) By the Routh–Hurwitz criterion for a cubic with $a_{1}>0$, the boundary of the stable region is reached either when $a_{3}=0$ (a real root crosses zero, λ=0) or when $a_{1}a_{2}-a_{3}=0$ with $a_{3}>0$ (a conjugate pair $\lambda =\pm i\sqrt{a_{2}}$ crosses the imaginary axis); these are mutually exclusive at a first crossing, and the affine (hence monotone) dependence in (i) selects whichever occurs at the smaller ξ. The frequency follows from substituting $\lambda =i\omega^{*}$ into the cubic, which gives $\omega^{*2}=a_{2}$.    □

**Remark** **5** (Application to the computational parameter set)**.** For Table 3 one has $A_{k}t-ps<0$ throughout the unstable band, so $a_{3}\left(k;\xi \right)$ stays positive and case (b) applies: the onset is oscillatory. The first admissible Neumann mode to lose stability is $k^{*}=5$, at $\xi_{c}\approx 12.48$ (the continuous-μ infimum reported in Section 7.3 is 12.47, the two agreeing to within the discrete mode spacing $\mu_{k+1}-\mu_{k}$). At this onset the critical mode has a marginal complex pair $\lambda =\pm i\;\omega^{*}$ with $\omega^{*}\approx 0.99$, the Hopf condition $a_{1}a_{2}-a_{3}=0$ holds exactly while $a_{3}\approx 4.68>0$, and consistently $\omega^{*}=\sqrt{a_{2}}$ and the third eigenvalue is real at $-a_{1}\approx -4.78$. All five quantities are checked in the onset-diagnostics block of Section 7.3 (computed values $\xi_{c}=12.479$, $k^{*}=5$, $\omega^{*}=0.989$, $a_{1}a_{2}-a_{3}=-1.5\times 10^{-11}$, $a_{3}=4.681$). This is the statement about the Hopf-type onset, and it is why the onset must be located from $\omega_{k}$ directly rather than from the constant-term threshold $\xi_{k}^{*}$. Note that this $k^{*}=5$ is the mode that first goes unstable at $\xi_{c}$; the dominant (fastest-growing) mode at the super-critical $\xi =1.6\;\xi_{c}$ used in Section 7.3 is instead k=6, and the two need not coincide.

By the Routh–Hurwitz criterion, mode *k* is stable if and only if

(41) $$a_{1}\left(k\right)>0,\;a_{2}\left(k\right)>0,\;a_{3}\left(k\right)>0,\;a_{1}\left(k\right)a_{2}\left(k\right)>a_{3}\left(k\right).$$

Because the diffusion and reaction contributions make $a_{1}\left(k\right)>0$ automatically, the loss of stability occurs through $a_{3}\left(k\right)=0$ (a stationary, Turing-type crossing) or through $a_{1}\left(k\right)a_{2}\left(k\right)=a_{3}\left(k\right)$ (an oscillatory, Hopf-type crossing); this mode-projection approach to diffusion-driven instability is classical in reaction–diffusion pattern formation [77]. The chemotactic sensitivity enters both $a_{2}\left(k\right)$ and $a_{3}\left(k\right)$ through the amplified coupling $r_{k}=\sigma_{1}/{\left(1+w^{*}\right)}^{2}+\xi v^{*}\mu_{k}$. Appendix B.5 gives an explicit constant-term destabilization threshold $\xi_{k}^{*}$ that is valid only when a sign condition holds; since the first crossing may instead be of Hopf type, the onset is most safely located from (39) and (40) directly, and that is the route used in the numerical experiments of Section 7.

The destabilizing coupling combines two effects. The recruitment sensitivity $\sigma_{1}/{\left(1+w^{*}\right)}^{2}$ measures how strongly local immune recruitment responds to the chemokine concentration, while the spatial term $\xi v^{*}\mu_{k}$ measures the chemotactic amplification of the immune response to a chemokine mode of wavenumber $\mu_{k}$. Because the latter grows with $\mu_{k}$, a sufficiently large chemotactic sensitivity destabilizes a band of finite wavelengths even when the homogeneous coexistence state is stable to spatially uniform perturbations. This is the mathematical mechanism behind the transition from homogeneous tumour–immune coexistence to spatially heterogeneous immune infiltration or exclusion patterns, which is verified numerically in Section 7.

## 7. Results (Numerical Experiments)

The numerical experiments in this section are organized around the theoretical results of Section 4 and Section 5. Rather than presenting simulations as illustrations, each experiment is designed to verify a specific analytical statement and to quantify the agreement between theory and computation. Concretely, we test the tumour-free threshold of Theorem 2 (Experiment 1), the coexistence construction of Proposition 4 (Experiment 2), the chemotaxis dispersion relation derived below (Experiment 3), the chemotaxis-driven onset of spatial heterogeneity (Experiment 4), the parametric structure of the stability boundary (Experiment 5), and the convergence, positivity, and residual consistency of the finite-volume scheme of Section 3 (Experiment 6).

All computations use the conservative finite-volume discretization of Section 3 with upwind chemotactic flux, discrete no-flux boundaries, and implicit (BDF) time stepping, on the one-dimensional domain Ω=(0,L) with L=10. Unless stated otherwise, the parameters are those of Table 3, for which the homogeneous coexistence equilibrium of Proposition 4 is

(42) $$\left(u^{*},v^{*},w^{*}\right)=\left(0.5311,\;0.4689,\;0.3900\right),$$

obtained as the unique interior root of F(u)=0 in (36). This equilibrium is stable to spatially homogeneous perturbations (the eigenvalues of the reaction Jacobian are −1.302 and −0.371±0.211i), so any instability reported below is genuinely chemotaxis- and wavenumber-driven.

The linear-stability experiments use the mode-wise stability matrix $M_{k}$ of (38), derived in Section 6 by linearizing about the homogeneous coexistence equilibrium and projecting onto the Neumann Laplacian eigenbasis $-\Delta \phi_{k}=\mu_{k}\phi_{k}$ (so that $\mu_{k}={\left(k\pi /L\right)}^{2}$ in one dimension). We recall that the chemotactic sensitivity enters $M_{k}$ only through the (v,w) entry as $+\xi v^{*}\mu_{k}$, that the dispersion relation is ωk(ξ)=maxjReλj(Mk), and that the critical sensitivity $\xi_{c}$ is the smallest ξ for which $\max_{k\geq 1}\omega_{k}\left(\xi \right)=0$.

### 7.1. Experiment 1: Tumour-Free Threshold

We use this experiment to verify Theorem 2: the tumour-free equilibrium $E_{0}=\left(0,\sigma_{0}/\delta ,0\right)$ is linearly stable for $\sigma_{0}>\delta$ and unstable for $\sigma_{0}<\delta$, with linear tumour growth rate $1-\sigma_{0}/\delta$ independent of chemotaxis.

We fix δ=0.30 and take a small tumour seed $u_{0}=10^{-3}\left(1+0.3\cos\left(2\pi x/L\right)\right)$ on the tumour-free background $v_{0}=\sigma_{0}/\delta$, $w_{0}=0$, with ξ=0. Two regimes are compared: the stable case $\sigma_{0}=0.50>\delta$ and the invasion case $\sigma_{0}=0.10<\delta$.

**Figure 4:**
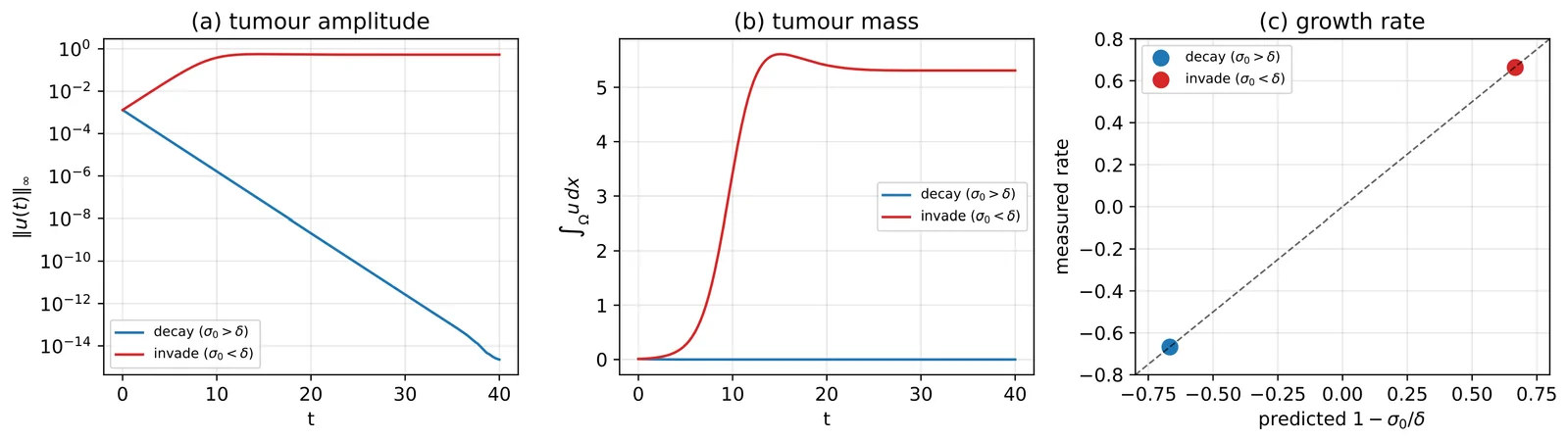
Experiment 1. (**a**) Tumour amplitude ${∥u\left(t\right)∥}_{\infty }$ for the decay regime $\sigma_{0}>\delta$ and the invasion regime $\sigma_{0}<\delta$; (**b**) tumour mass $\int_{\Omega }u\;dx$; (**c**) measured versus predicted growth rate $1-\sigma_{0}/\delta$ (dashed line is identity). The measured rates agree with Theorem 2.

Figure 4 reports ${∥u\left(t\right)∥}_{\infty }$, the tumour mass $\int_{\Omega }u\;dx$, and the measured exponential rate against the predicted value $1-\sigma_{0}/\delta$. In the stable regime the tumour decays to machine zero at the rate −0.6677 (predicted −0.6667); in the invasion regime it grows at 0.6635 (predicted 0.6667) and saturates at the coexistence value $u^{*}=0.531$ from (42). The measured rates lie on the diagonal of Figure 4c, confirming the threshold and the predicted growth rate to within the time-stepping tolerance.

### 7.2. Experiment 2: Coexistence Equilibrium Verification

We use this experiment to verify Proposition 4: starting from generic positive data, the dynamics (with ξ=0, below any patterning threshold) converge to the homogeneous coexistence state determined by the scalar equation $F(u^{*})=0$, with $v^{*}=1-u^{*}$ and $w^{*}=u^{*}\left[\alpha +\gamma \left(1-u^{*}\right)\right]/l$.

We integrate from spatially random nonnegative initial data $u_{0},v_{0},w_{0}\in \left[0.2,0.5\right]$ to t=200 and compare the numerical steady state with the analytical equilibrium (42).

**Figure 5:**
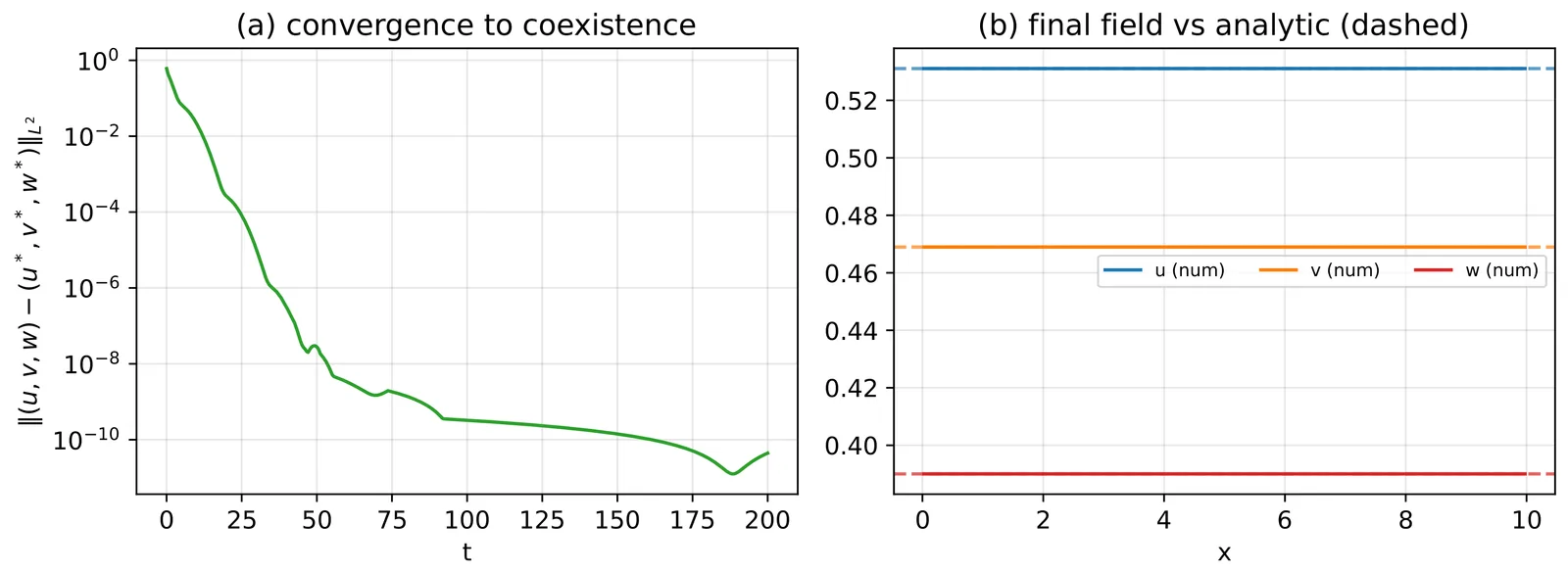
Experiment 2. (**a**) Convergence of the solution to the analytical coexistence equilibrium in $L^{2}$; (**b**) final numerical fields (solid) versus the analytical values $\left(u^{*},v^{*},w^{*}\right)$ of (42) (dashed).

Figure 5 shows the monotone convergence of $∥\left(u,v,w\right)-\left(u^{*},v^{*},w^{*}\right){∥}_{L^{2}}$ to $4.4\times 10^{-11}$, and the final spatial fields coincide with the analytical equilibrium (dashed) to the same order. The componentwise errors are $1.1\times 10^{-11}$, $4.7\times 10^{-12}$, and $7.7\times 10^{-12}$ for *u*, *v*, *w* respectively, confirming both the existence construction and the closed-form expressions for $v^{*}$ and $w^{*}$.

### 7.3. Experiment 3: Dispersion Relation and Unstable Modes

We use this experiment to verify the dispersion relation associated with (38): the eigenvalues of $M_{k}$ predict which spatial modes grow, the dominant wavenumber, and the per-mode growth rates measured from the full nonlinear simulation.

About the coexistence equilibrium (42) we compute maxjReλj(Mk) as a function of the wavenumber index *k* for several values of ξ. The smallest sensitivity at which a finite-*k* mode becomes neutral is $\xi_{c}\approx 12.47$. For $\xi =1.6\;\xi_{c}$ we then integrate the nonlinear system from a small random perturbation of the equilibrium, extract the discrete-cosine (Neumann-mode) amplitudes of *v*, and fit their exponential growth rate during the linear phase.

**Figure 6:**
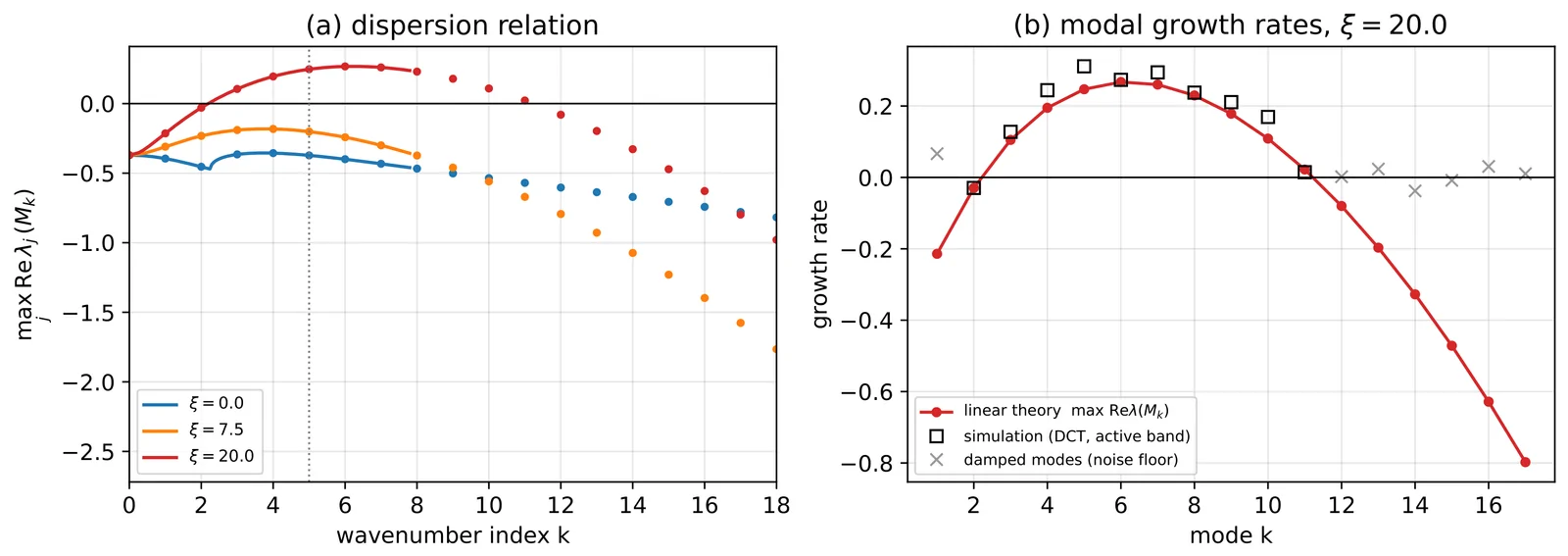
Experiment 3. (**a**) Dispersion relation maxjReλj(Mk) versus wavenumber index *k* for $\xi \in \{0,\;0.6\;\xi_{c},\;1.6\;\xi_{c}\}$ (curves: continuous μ; markers: admissible Neumann modes). (**b**) Linear-theory modal growth rates (line) versus rates measured from the nonlinear simulation via the discrete cosine transform (squares: active band; crosses: damped modes at the noise floor) for $\xi =1.6\;\xi_{c}$.

Figure 6a shows that the homogeneous and weakly chemotactic cases are stable for all *k*, while for $\xi =1.6\;\xi_{c}$ a band of intermediate wavenumbers becomes unstable, with maximal growth near $k^{*}=6$. Panel (b) compares the linear-theory growth rates with the simulated modal growth rates: in the active (near-marginal and unstable) band the agreement is excellent, with correlation 0.980, and the dominant mode matches in both location ($k^{*}=6$) and rate (predicted 0.267, measured 0.273). Strongly damped modes saturate at the nonlinear noise floor rather than following their large negative linear rates, as expected, since linear theory governs only the growing band.

### 7.4. Experiment 4: Chemotaxis-Driven Pattern Formation

We use this experiment to verify that $\xi_{c}$ separates spatially homogeneous behaviour from spatially heterogeneous tumour–immune organization: convergence to the homogeneous coexistence state for $\xi <\xi_{c}$, marginal small-amplitude structure near $\xi_{c}$, and a sustained pattern for $\xi >\xi_{c}$.

Starting from the same perturbed coexistence data, we integrate to t=120 for $\xi =0.7\;\xi_{c}$ (sub-critical), $\xi \approx \xi_{c}$ (onset), and $\xi =1.8\;\xi_{c}$ (super-critical), tracking the spatial amplitude $\max_{x}u-\min_{x}u$.

**Figure 7:**
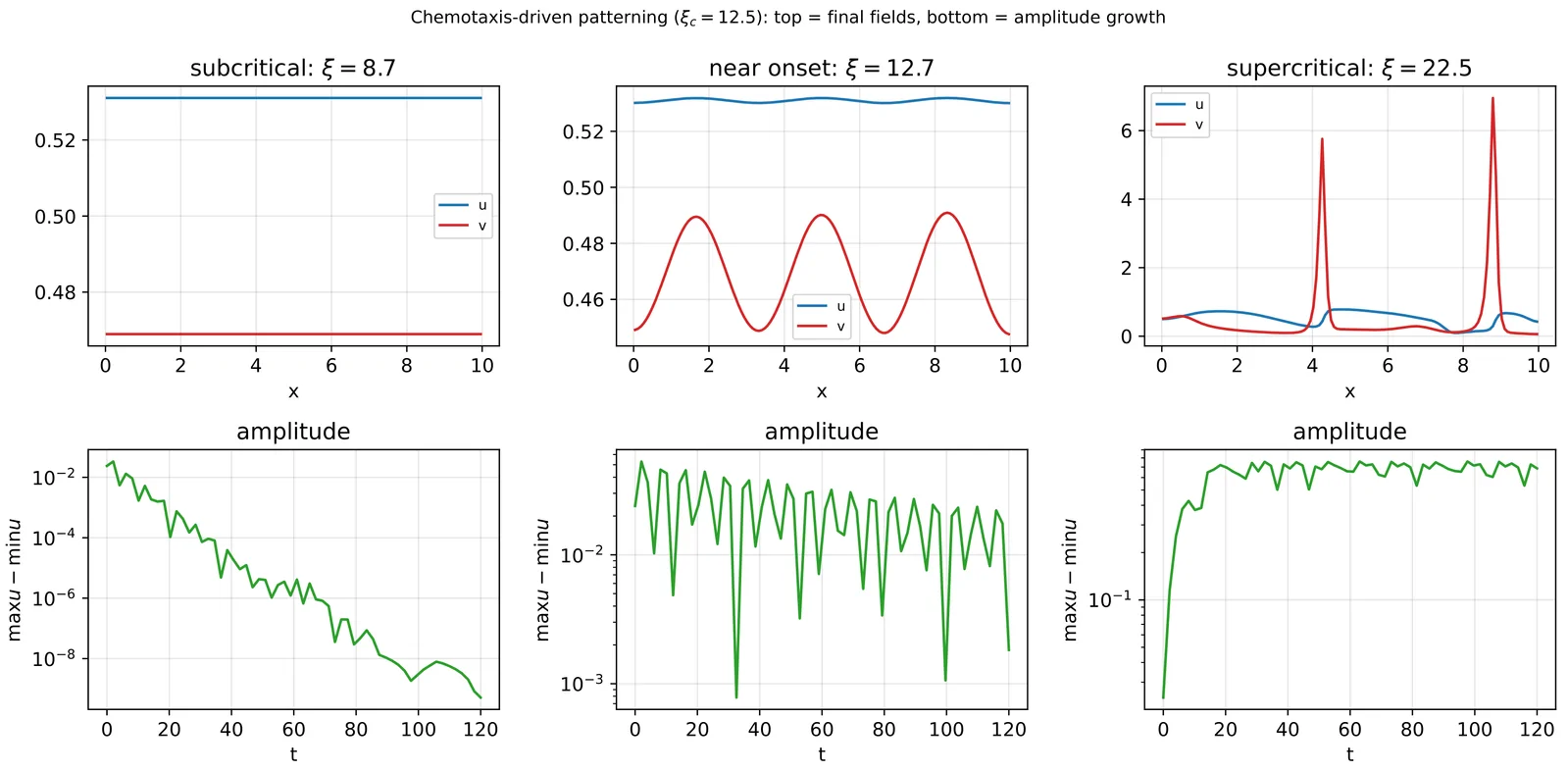
Experiment 4. (**Top row**): final tumour (*u*) and immune (*v*) fields for sub-critical, near-critical, and super-critical chemotactic sensitivity. (**Bottom row**): the corresponding spatial amplitude $\max_{x}u-\min_{x}u$ versus time. The transition homogeneous → onset → heterogeneity occurs across $\xi_{c}\approx 12.5$.

Figure 7 confirms the predicted transition. For $\xi =0.7\;\xi_{c}$ the amplitude decays to $∼10^{-9}$ and the fields return to homogeneity; near $\xi_{c}$ a small steady modulation of amplitude $\approx 5\times 10^{-3}$ persists; for $\xi =1.8\;\xi_{c}$ the system develops a large-amplitude pattern in which the immune density forms sharp aggregation peaks at chemokine maxima (maxu−minu≈0.69, maxv−minv≈6.9) with the tumour correspondingly depleted there. The minimum of every component remains strictly positive throughout (e.g., minu≈0.10, minv≈0.06 in the super-critical run), consistent with the positivity property of Theorem 1 and the positivity-preserving design of the scheme.

### 7.5. Experiment 5: Two-Parameter Sensitivity Maps

We use this experiment to characterize how the patterning threshold depends on the model parameters by mapping the stability boundary in the planes $\left(\xi ,\sigma_{1}\right)$, (ξ,δ), and $\left(\xi ,d_{2}\right)$. This directly addresses the sensitivity analysis of the chemotaxis-driven instability.

For each point of a parameter grid we recompute the coexistence equilibrium from $F(u^{*})=0$ and evaluate maxk≥1maxjReλj(Mk) over the admissible Neumann modes. The zero level set of this quantity is the onset curve.

**Figure 8:**
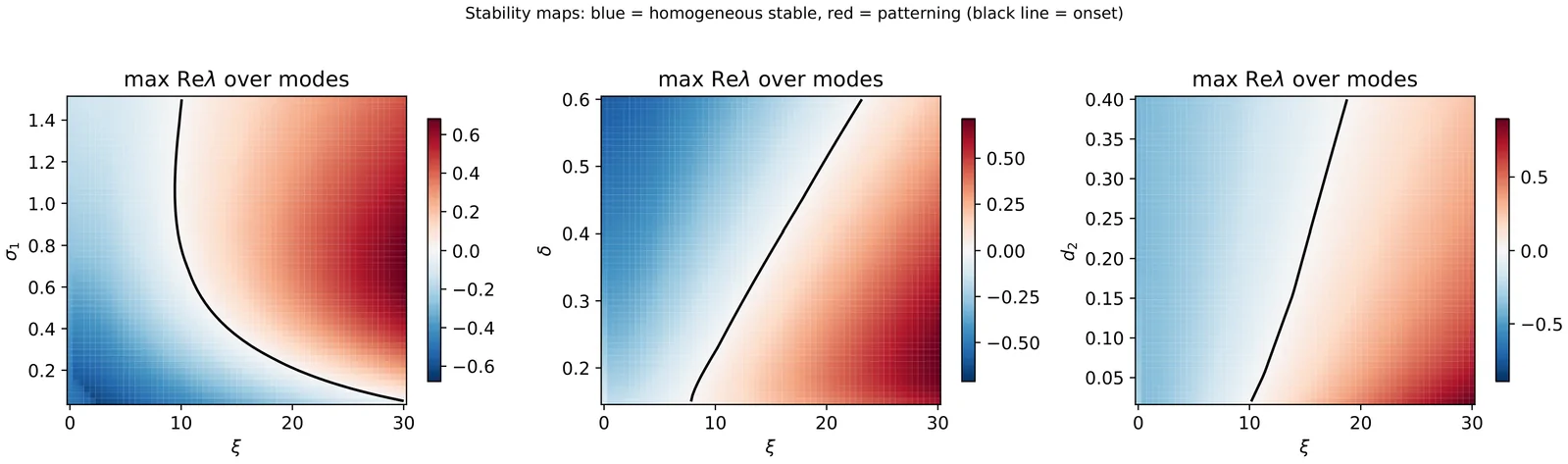
Experiment 5. Stability maps of maxk≥1maxjReλj(Mk) in the planes $\left(\xi ,\sigma_{1}\right)$, (ξ,δ), and $\left(\xi ,d_{2}\right)$. Blue: homogeneous coexistence stable; red: chemotaxis-driven patterning; black curve: onset (Reλ=0). The equilibrium is recomputed at every grid point.

Figure 8 shows the three stability maps. The instability region (positive growth, red) expands as the chemokine-induced recruitment $\sigma_{1}$ increases, so that stronger recruitment lowers the critical sensitivity $\xi_{c}$. Conversely, increasing the immune decay δ or the immune diffusion $d_{2}$ shrinks the unstable region and raises $\xi_{c}$: immune turnover and random immune motility are both stabilizing, since they damp the spatial immune aggregation that drives the instability. The monotone, smooth dependence of the onset curve on each parameter quantifies the competition between chemotactic aggregation and the stabilizing effects of immune loss and diffusion.

### 7.6. Experiment 6: Grid Convergence and Residual Certification

We use this experiment to certify that the computed patterns are properties of the PDE system and not artifacts of the discretization by demonstrating grid convergence, discrete positivity, and small post hoc PDE residuals (21)–(23).

In a mildly super-critical regime ($\xi =1.3\;\xi_{c}$, smooth low-amplitude pattern) we integrate to t=60 on grids N∈{64,128,256,512}. We report the successive differences $∥U_{h}-U_{h/2}{∥}_{L^{2}}$ (fine fields averaged to the coarse grid), the running minimum of each component, and the residual norms $∥R_{u}∥,∥R_{v}∥,∥R_{w}∥$ evaluated on the trajectory.

**Figure 9:**
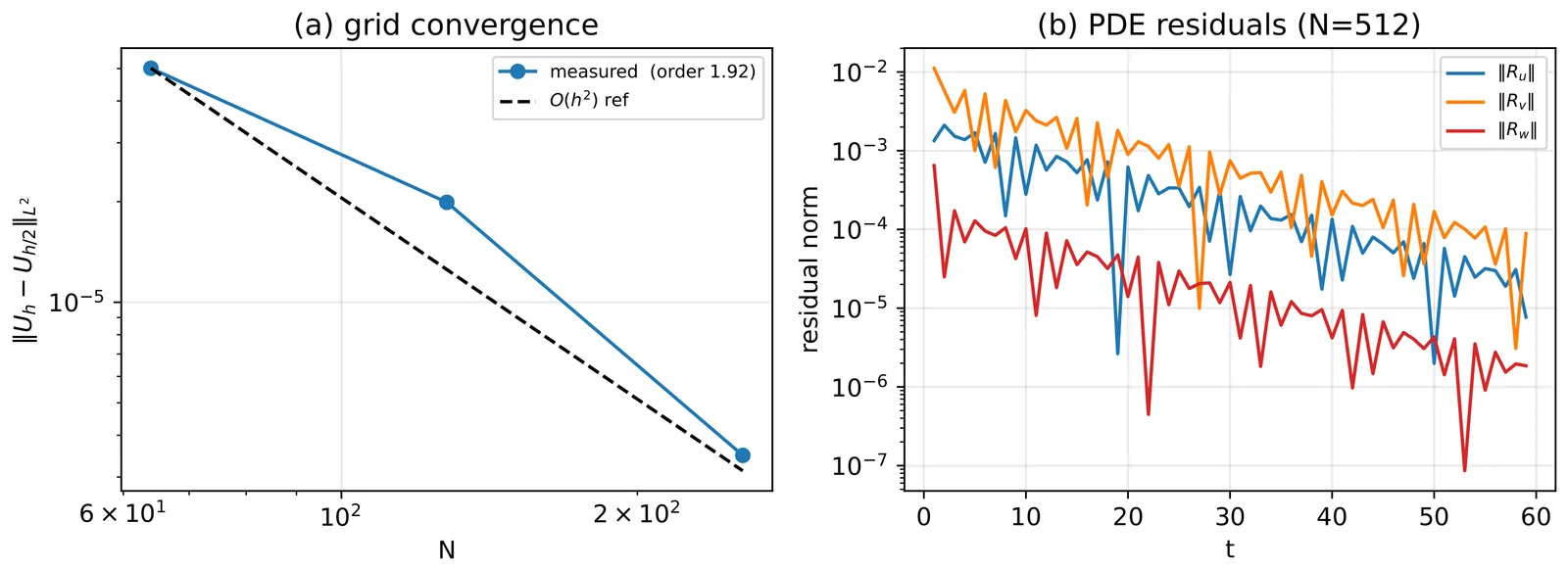
Experiment 6. (**a**) Grid convergence $∥U_{h}-U_{h/2}{∥}_{L^{2}}$ versus *N* (observed order 1.92; dashed $O(h^{2})$ reference). (**b**) Post hoc partial-differential-equation (PDE) residual norms $∥R_{u}∥,∥R_{v}∥,∥R_{w}∥$ versus time on the finest grid.

Figure 9a shows that $∥U_{h}-U_{h/2}{∥}_{L^{2}}$ decreases from $5.0\times 10^{-5}$ to $3.5\times 10^{-6}$ as *N* increases, an observed convergence order of 1.92. The order is close to two because the diffusion and reaction terms are second-order accurate and dominate here; the first-order upwind chemotactic flux reduces the order toward one only in strongly chemotaxis-dominated regimes with steep immune gradients. The running minimum of each component remains positive on every grid (uniformly ≈0.389, consistent with the small pattern amplitude), confirming discrete positivity. Figure 9b shows that the PDE residuals remain small and decay in time (maxima $∥R_{u}∥\approx 2\times 10^{-3}$, $∥R_{v}∥\approx 1\times 10^{-2}$, $∥R_{w}∥\approx 6\times 10^{-4}$), certifying that the computed solution is a faithful discrete realization of (4). Together the convergence, positivity, and residual diagnostics validate the spatial patterns reported in Experiments 3 and 4.

### 7.7. Experiment 7: Two-Dimensional Pattern and Immune-Infiltration Foci

**Figure 10:**
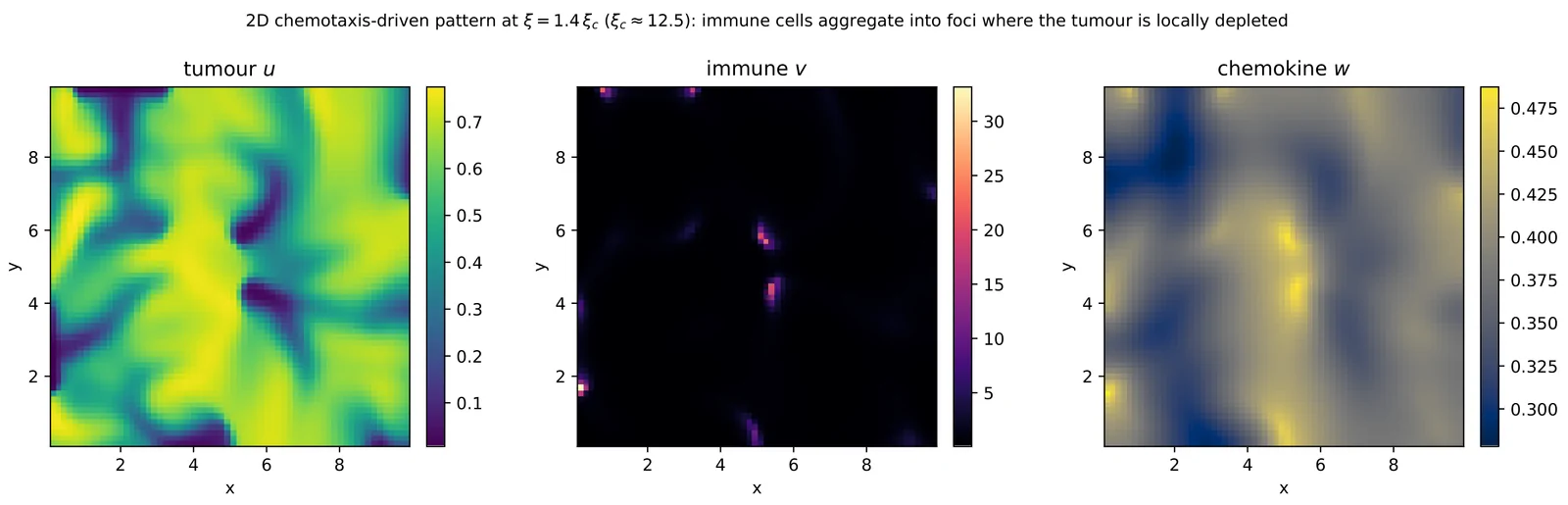
Experiment 7. Two-dimensional chemotaxis-driven pattern at $\xi =1.4\;\xi_{c}$ ($\xi_{c}\approx 12.5$) on $\Omega ={\left(0,10\right)}^{2}$, shown at t=80. (**Left**): tumour *u* (labyrinthine network of tumour-rich plateaus and depleted channels); (**centre**): immune *v* (discrete aggregation foci, peak ≈33 against baseline $v^{*}\approx 0.47$); (**right**): chemokine *w* (smooth field whose maxima coincide with the immune foci). Immune effectors concentrate into infiltration hot-spots at chemokine maxima where the tumour is locally cleared. Discrete positivity is preserved (min>0).

To illustrate the biologically relevant regime, we integrate the full system on the square $\Omega ={\left(0,10\right)}^{2}$ with the two-dimensional finite-volume scheme of Section 3.6, using the CFL-limited explicit stepper of Section 3.5 (which guarantees discrete positivity, see Proposition 1). Starting from a small perturbation of the coexistence equilibrium (42) at $\xi =1.4\;\xi_{c}$, the solution self-organizes into the pattern of Figure 10: the immune density condenses into sharp aggregation *foci* located at local maxima of the chemokine field, where the tumour is correspondingly depleted, while intervening regions remain immune-poor and tumour-rich. The minimum of every component stays strictly positive throughout ($\min\left(u,v,w\right)\approx 1.6\times 10^{-3}>0$), consistent with Proposition 1. This is the two-dimensional, spatially explicit counterpart of the “hot” (infiltrated) versus “cold” (excluded) dichotomy: chemokine-directed chemotaxis concentrates effector cells into discrete infiltration hot-spots rather than distributing them uniformly.

### 7.8. Summary of Verification

Table 4 collects the quantitative agreement between theory and computation. Across all six experiments, the simulations reproduce the analytical thresholds and equilibria to within solver tolerance and confirm the chemotaxis-driven origin of the spatial patterns, while the positivity, mass-balance, and residual diagnostics certify the reliability of the numerical results.

## 8. Conclusions

We have analyzed a minimal but mechanistically complete reaction–diffusion–chemotaxis model of tumour–immune–chemokine dynamics in which a tumour-produced chemokine both recruits immune effector cells and directs their chemotactic migration. After non-dimensionalization we established local classical solvability, invariance of the nonnegative cone, a uniform tumour-density bound, and global-in-time immune and chemokine mass estimates, and we delineated precisely why these estimates fall short of global $L^{\infty }$-boundedness for the full three-component chemotaxis system.

On the level of equilibria, the tumour-free state is governed by the immune control index $\sigma_{0}/\delta$, with linear stability for $\sigma_{0}>\delta$ and invasion for $\sigma_{0}<\delta$, and coexistence reduces to a single scalar equation $F(u^{*})=0$. Linearizing about coexistence and projecting onto the Neumann eigenbasis yields the stability matrix $M_{k}$ in which the chemotactic sensitivity appears only through the wavenumber-amplified coupling $\xi v^{*}\mu_{k}$. Through the Routh–Hurwitz analysis this coupling drives a finite-wavelength instability above a critical sensitivity $\xi_{c}$, which for the parameters studied here is an oscillatory (Hopf-type) crossing rather than a stationary one.

The numerical contribution is a conservative finite-volume scheme with upwind chemotactic flux, discrete no-flux boundaries, and positivity, mass-balance, and residual diagnostics. Rather than presenting simulations as illustrations, we organized the experiments around the explicit verification of each theoretical result. The simulations reproduced the tumour-free growth rate $1-\sigma_{0}/\delta$ and threshold, recovered the analytical coexistence equilibrium to solver tolerance, matched the dispersion relation and the dominant unstable wavenumber, exhibited the predicted homogeneous-to-heterogeneous transition across $\xi_{c}$, mapped the stability boundary in three parameter planes, and passed grid convergence and residual certification while preserving discrete positivity.

Several directions remain open. A global boundedness theory for the full system would require additional structure, such as small chemotactic sensitivity, stronger logistic damping, signal-dependent desensitization, or dimension-dependent regularity estimates. The weakly nonlinear analysis of the Hopf-type onset, the classification of the emerging spatio-temporal patterns in two and three dimensions, and the incorporation of treatment terms (immunotherapy, chemotherapy) are natural extensions of the present framework.

## Figures and Tables

**Table 1:** Dimensional parameters with representative biological orders of magnitude. Ranges are indicative of solid-tumour tissue; cell motilities are effective (random-motility) coefficients from intravital imaging, chemokine transport from tissue diffusion measurements, and kinetic rates from validated tumour–immune models. Values are used only to fix orders of magnitude, not to model a specific tumour.

Symbol	Meaning	Representative Estimate	Source
$d_{T}$	tumour-cell random motility	$∼10^{-2}$–$1\;\mu m^{2}\;s^{-1}$	(weakly motile)
$d_{E}$	immune-cell motility	∼1–$70\;\mu m^{2}\;\min^{-1}$ (∼0.02–$1\;\mu m^{2}\;s^{-1}$)	[70]
$d_{A}$	chemokine diffusion	∼10–$100\;\mu m^{2}\;s^{-1}$ ($10^{-7}$–$10^{-6}\;{\mathrm{cm}}^{2}\;s^{-1}$)	[71]
*r*	tumour growth rate	∼0.1–$0.5\;{\mathrm{day}}^{-1}$ (doubling days–weeks)	[72]
*K*	tumour carrying capacity	$∼10^{8}$–$10^{9}\;\mathrm{cells}\;{\mathrm{cm}}^{-3}$	tissue density
κ	immune killing rate	lysis-based, $O\left(10^{-1}\right)\;{\mathrm{day}}^{-1}$ per effector density	[72]
χ	chemotactic sensitivity	$∼10^{-8}$–$10^{-6}\;{\mathrm{cm}}^{2}\;s^{-1}$ per unit signal	(chemotaxis assays)
$s_{0}$	baseline immune influx	$∼10^{-2}\;{\mathrm{day}}^{-1}$	[72]
$s_{1}$	max. chemokine recruitment	comparable to/above $s_{0}$	[72]
η	half-saturation constant	signal-dependent (nM range)	(receptor affinity)
μ	immune-cell loss rate	∼0.01–$0.2\;{\mathrm{day}}^{-1}$	[72]
ρ	tumour-induced immune loss	O(μ)	[72]
$\alpha_{T},\alpha_{C}$	chemokine production	per-cell secretion, $O\left(10^{-4}\right)\;\mathrm{ng}\;{\mathrm{cells}}^{-1}{\mathrm{day}}^{-1}$	[73,74]
λ	chemokine degradation	∼0.03–$1\;h^{-1}$ (half-life min–h)	[71]
*L*	reference tissue length	$∼10^{2}\;\mu m^{-1}$–1cm	(signalling niche)

**Table 2:** Non-dimensional parameters in (4).

Symbol	Definition	Interpretation
$d_{1}$	$d_{T}/\left(rL^{2}\right)$	tumour diffusion relative to growth
$d_{2}$	$d_{E}/\left(rL^{2}\right)$	immune diffusion relative to tumour growth
$d_{3}$	$d_{A}/\left(rL^{2}\right)$	chemokine diffusion relative to tumour growth
ξ	$\chi \eta /(rL^{2})$	chemotactic sensitivity
$\sigma_{0}$	$\kappa s_{0}/r^{2}$	baseline immune supply
$\sigma_{1}$	$\kappa s_{1}/r^{2}$	chemokine-induced immune recruitment
δ	μ/r	immune decay relative to tumour growth
β	ρK/r	tumour-induced immune loss strength
α	$\alpha_{T}K/\left(r\eta \right)$	tumour-driven chemokine production
γ	$\alpha_{C}K/\left(\kappa \eta \right)$	interaction-driven chemokine production
*ℓ*	λ/r	chemokine degradation relative to tumour growth

**Table 3:** Baseline non-dimensional parameters used in the numerical experiments in Section 7. Experiment 1 instead varies $\sigma_{0}$ with δ=0.30 fixed; Experiment 5 varies the indicated pairs.

$d_{1}$	$d_{2}$	$d_{3}$	$\sigma_{0}$	$\sigma_{1}$	δ
0.01	0.10	1.00	0.10	0.50	0.30
β	α	γ	*ℓ*	*L*	
0.40	0.50	0.50	1.00	10	

**Table 4:** Quantitative verification of the theoretical results.

Experiment	Theoretical Prediction	Numerical Result
1 (decay)	rate $1-\sigma_{0}/\delta =-0.6667$	−0.6677
1 (invasion)	rate $1-\sigma_{0}/\delta =+0.6667$	+0.6635
2	$\left(u^{*},v^{*},w^{*}\right)=\left(0.5311,0.4689,0.3900\right)$	error $4.4\times 10^{-11}$
3	dominant mode $k^{*}=6$, rate 0.267	$k^{*}=6$, rate 0.273 (corr. 0.980)
4	onset at $\xi_{c}\approx 12.47$	decay/onset/pattern across $\xi_{c}$
5	$\sigma_{1}↑$ destabilizes; $\delta ,d_{2}↑$ stabilize	confirmed monotone onset curves
6	convergent, positive, consistent	order 1.92, min>0, residuals $≲10^{-2}$

## Data Availability

All simulations were implemented in Python 3 using NumPy and SciPy; the stiff semi-discrete systems were advanced with the implicit BDF integrator of scipy.integrate.solve_ivp, and the two-dimensional explicit runs used the CFL-limited forward-Euler stepper of Section 3.5. Figures were produced with Matplotlib (version number: 3.11.1). The complete source code, together with the scripts that regenerate every figure and table in this paper, is openly available at https://github.com/Louis-shuo-wang/second-Bioengineering.git (accessed on 11 August 2026).

## Abbreviations

The following abbreviations are used in this manuscript:

PDE	Partial Differential Equation
BDF	Backward Differentiation Formula

## Appendix A. Proofs of Technical Estimates

This appendix collects the technical arguments supporting the local solvability, positivity, and a priori estimates used in Section 4. The goal is not to prove a full global boundedness theorem for the three-component chemotaxis system, but rather to record the estimates that are sufficient for the equilibrium, stability, and numerical verification results developed in the main text. We consider the non-dimensional system (4) posed in a bounded smooth domain $\Omega \subset R^{d}$ with the no-flux boundary conditions (5). The initial data are assumed to satisfy

$$u_{0},v_{0},w_{0}\geq 0\;\mathrm{in}\;\bar{\Omega }.$$

### Appendix A.1. Normal Parabolicity, Local Solvability, and Positivity

Writing the principal part of (4) in divergence form,

$$\partial_{t}U=\nabla \cdot \left(A(U)\nabla U\right)+R\left(U\right),\;U={\left(u,v,w\right)}^{⊤},$$

gives

$$A\left(U\right)=\left(\begin{array}{ccc} d_{1} & 0 & 0\\ 0 & d_{2} & -\xi \;v\\ 0 & 0 & d_{3} \end{array}\right).$$

For every $\zeta \in R^{d}\setminus \left\{0\right\}$,

$$\sigma \;\left({\left|\zeta \right|}^{2}A\left(U\right)\right)=\{d_{1}{\left|\zeta \right|}^{2},d_{2}{\left|\zeta \right|}^{2},d_{3}{\left|\zeta \right|}^{2}\}.$$

Consequently, with $d_{*}:=\min\left\{d_{1},d_{2},d_{3}\right\}>0$,

minλ∈σ(|ζ|2A(U))Reλ≥d*|ζ|2.

This is the normal-ellipticity condition. The conclusion remains valid when two diffusivities coincide, even though the (v,w) block may then have a nontrivial Jordan form. On every set |v|≤M, the coefficients and the corresponding sectoriality constants are uniform.

The boundary conditions are compatible with this normally elliptic principal part. Indeed, $\partial_{n}w=0$ and $d_{2}\partial_{n}v-\xi v\partial_{n}w=0$ imply $\partial_{n}v=0$, so the no-flux conditions are equivalent, for classical solutions, to componentwise homogeneous Neumann conditions. The associated boundary operator satisfies the standard complementing condition. Moreover, A and the reaction map are smooth on the open phase domain

$$O:=R^{2}\times \left(-1,\infty \right).$$

Thus, on a smooth bounded domain and for sufficiently regular compatible initial data, standard quasilinear parabolic theory [76] yields a unique maximal classical solution.

We next prove invariance of the nonnegative cone. Fix $T<T_{\max}$. Classical regularity implies that u,v,w and ∇w are bounded on $\left[0,T\right]\times \bar{\Omega }$. By continuity, it is enough initially to work on a time interval on which w>−1/2. Define

$$u_{-}:=\max\left\{-u,0\right\},\;v_{-}:=\max\left\{-v,0\right\},\;w_{-}:=\max\left\{-w,0\right\}.$$

Testing the immune equation in conservative form by $-v_{-}$ and using the no-flux boundary condition gives

$$\begin{array}{cc} \frac{1}{2}\frac{d}{dt}{∥v_{-}∥}_{L^{2}}^{2}= & -d_{2}{∥\nabla v_{-}∥}_{L^{2}}^{2}+\xi \int_{\Omega }v_{-}\nabla v_{-}\cdot \nabla w\;dx\\ & -\int_{\Omega }\left(\delta +\beta u\right)v_{-}^{2}\;dx-\int_{\Omega }\left(\sigma_{0}+\sigma_{1}\frac{w}{1+w}\right)v_{-}\;dx. \end{array}$$

Young’s inequality and boundedness of *u* and ∇w imply

$$\frac{1}{2}\frac{d}{dt}∥v_{-}{∥}_{L^{2}}^{2}+\frac{d_{2}}{2}∥\nabla v_{-}{∥}_{L^{2}}^{2}\leq C_{T}{∥v_{-}∥}_{L^{2}}^{2}-\int_{\Omega }\left(\sigma_{0}+\sigma_{1}\frac{w}{1+w}\right)v_{-}\;dx.$$

On {w<0}, because w>−1/2,

$$\left|\frac{w}{1+w}\right|\leq 2w_{-},$$

and hence

$$-\left(\sigma_{0}+\sigma_{1}\frac{w}{1+w}\right)v_{-}\leq 2\sigma_{1}w_{-}v_{-}\leq \sigma_{1}\left(w_{-}^{2}+v_{-}^{2}\right).$$

Therefore

$$\frac{d}{dt}{∥v_{-}∥}_{L^{2}}^{2}\leq C_{T}\left(∥v_{-}{∥}_{L^{2}}^{2}+{∥w_{-}∥}_{L^{2}}^{2}\right).$$

Testing the *u*- and *w*-equations by $-u_{-}$ and $-w_{-}$, respectively, gives

$$\frac{d}{dt}∥u_{-}{∥}_{L^{2}}^{2}\leq C_{T}{∥u_{-}∥}_{L^{2}}^{2}$$

and

$$\frac{d}{dt}{∥w_{-}∥}_{L^{2}}^{2}\leq C_{T}\left(∥u_{-}{∥}_{L^{2}}^{2}+∥v_{-}{∥}_{L^{2}}^{2}+{∥w_{-}∥}_{L^{2}}^{2}\right).$$

For the latter estimate we use the boundedness of *u* and *v* and

$${\left(uv\right)}_{-}\leq \left|v\right|u_{-}+\left|u\right|v_{-}.$$

Adding the three inequalities and applying Gronwall’s lemma yields

$$∥u_{-}{\left(t\right)∥}_{L^{2}}^{2}+∥v_{-}{\left(t\right)∥}_{L^{2}}^{2}+{∥w_{-}\left(t\right)∥}_{L^{2}}^{2}=0$$

because all three negative parts vanish initially. A continuation argument then extends the conclusion to every $T<T_{\max}$. Thus

u(t,x)≥0,v(t,x)≥0,w(t,x)≥0

throughout the classical solution’s interval of existence.

### Appendix A.2. Tumour-Density Bound

We prove Proposition 2. Since v≥0, the first equation of (4) satisfies

$$u_{t}=d_{1}\Delta u+u\left(1-u-v\right)\leq d_{1}\Delta u+u\left(1-u\right).$$

Let y(t) solve the scalar logistic equation

$$y^{\prime }=y\left(1-y\right),\;y\left(0\right)={∥u_{0}∥}_{L^{\infty }\left(\Omega \right)}.$$

The comparison principle under homogeneous Neumann boundary conditions gives

$$0\leq u\left(t,x\right)\leq y\left(t\right)\;\mathrm{for}\;0<t<T_{\max},\;x\in \bar{\Omega }.$$

The explicit solution of the logistic equation satisfies

y(t)≤max{1,y(0)}.

Therefore

(A1) $$0\leq u\left(t,x\right)\leq M_{u}:=\max\{1,∥u_{0}{∥}_{L^{\infty }\left(\Omega \right)}\}\;\mathrm{for}\;0<t<T_{\max}.$$

### Appendix A.3. Immune Mass Estimate

We prove the first estimate in Proposition 3. Integrating the *v*-equation in (4) over Ω gives

$$\frac{d}{dt}\int_{\Omega }v\;dx=d_{2}\int_{\Omega }\Delta v\;dx-\xi \int_{\Omega }\nabla \cdot \left(v\nabla w\right)\;dx+\int_{\Omega }\left(\sigma_{0}+\sigma_{1}\frac{w}{1+w}-\delta v-\beta uv\right)dx.$$

The diffusion and chemotaxis integrals vanish because of the no-flux condition:

$$\int_{\Omega }\nabla \cdot \left(d_{2}\nabla v-\xi v\nabla w\right)\;dx=\int_{\partial \Omega }\left(d_{2}\nabla v-\xi v\nabla w\right)\cdot n\;dS=0.$$

Since u,v,w≥0 and

$$0\leq \frac{w}{1+w}\leq 1,$$

we obtain

(A2) $$\frac{d}{dt}\int_{\Omega }v\;dx\leq \left|\Omega \right|\left(\sigma_{0}+\sigma_{1}\right)-\delta \int_{\Omega }v\;dx.$$

Solving the scalar differential inequality yields

(A3) $$\int_{\Omega }v\left(t,x\right)\;dx\leq e^{-\delta t}\int_{\Omega }v_{0}\left(x\right)\;dx+\frac{\left|\Omega |(\sigma_{0}+\sigma_{1}\right)}{\delta }\left(1-e^{-\delta t}\right).$$

Consequently,

(A4) $$\underset{0<t<T_{\max}}{\sup}\int_{\Omega }v\left(t,x\right)\;dx\leq C_{v},$$

where

$$C_{v}:=\max\left\{\int_{\Omega }v_{0}\left(x\right)\;dx,\;\frac{\left|\Omega |(\sigma_{0}+\sigma_{1}\right)}{\delta }\right\}.$$

### Appendix A.4. Chemokine Mass Estimate

We now prove the chemokine mass estimate. Integrating the *w*-equation in (4) over Ω and using the no-flux boundary condition gives

$$\frac{d}{dt}\int_{\Omega }w\;dx=\int_{\Omega }\left(\alpha u+\gamma uv-lw\right)dx.$$

Using the tumour-density bound (A1), we obtain

$$\int_{\Omega }\alpha u\;dx\leq \alpha M_{u}\left|\Omega \right|,\;\int_{\Omega }\gamma uv\;dx\leq \gamma M_{u}\int_{\Omega }v\;dx.$$

Therefore

(A5) $$\frac{d}{dt}\int_{\Omega }w\;dx\leq \alpha M_{u}\left|\Omega \right|+\gamma M_{u}\int_{\Omega }v\;dx-l\int_{\Omega }w\;dx.$$

By (A4),

$$\int_{\Omega }v\left(t,x\right)\;dx\leq C_{v}\;\mathrm{for}\;\mathrm{all}\;t<T_{\max}.$$

Thus

$$\frac{d}{dt}\int_{\Omega }w\;dx\leq \alpha M_{u}\left|\Omega \right|+\gamma M_{u}C_{v}-l\int_{\Omega }w\;dx.$$

Solving this scalar differential inequality gives

(A6) $$\int_{\Omega }w\left(t,x\right)\;dx\leq e^{-lt}\int_{\Omega }w_{0}\left(x\right)\;dx+\frac{\alpha M_{u}\left|\Omega \right|+\gamma M_{u}C_{v}}{l}\left(1-e^{-lt}\right).$$

Consequently,

(A7) $$\underset{0<t<T_{\max}}{\sup}\int_{\Omega }w\left(t,x\right)\;dx\leq C_{w},$$

where

$$C_{w}:=\max\left\{\int_{\Omega }w_{0}\left(x\right)\;dx,\;\frac{\alpha M_{u}\left|\Omega \right|+\gamma M_{u}C_{v}}{l}\right\}.$$

### Appendix A.5. Why These Estimates Do Not Imply Full Global Boundedness

The estimates derived above imply

$${∥u\left(t\right)∥}_{L^{\infty }\left(\Omega \right)}\leq M_{u}{,\;∥v\left(t\right)∥}_{L^{1}\left(\Omega \right)}\leq C_{v},\;{∥w\left(t\right)∥}_{L^{1}\left(\Omega \right)}\leq C_{w}$$

on the interval of classical existence. These estimates are sufficient for the basic biological interpretation of the model and for the equilibrium calculations in the main text. However, they do not by themselves imply

$$\underset{0<t<T_{\max}}{\sup}\left({∥v\left(t\right)∥}_{L^{\infty }\left(\Omega \right)}+{∥w\left(t\right)∥}_{L^{\infty }\left(\Omega \right)}\right)<\infty .$$

The reason is that the immune equation contains the chemotactic aggregation term

−ξ∇·(v∇w),

which can transfer spatial gradients of the chemokine field into concentration of the immune density. An $L^{1}$ bound on *v* and an $L^{1}$ bound on *w* do not control the gradients ∇w or the pointwise size of *v*. Thus a full global boundedness theorem would require additional structure, such as one or more of the following: small chemotactic sensitivity, strong logistic damping, signal-dependent chemotactic desensitization, entropy or Lyapunov estimates, dimension-dependent regularity arguments, or stronger a priori control of ∇w. For this reason, the main text states local classical well-posedness, positivity, tumour-density boundedness, and mass estimates, but does not claim global $L^{\infty }$-boundedness for the full three-component chemotaxis system in arbitrary dimension.

## Appendix B. Details of the Routh–Hurwitz Calculation

This appendix gives the algebra leading to the mode-wise stability criterion used in Section 6 and Section 7. We linearize the non-dimensional system (5) about a spatially homogeneous coexistence equilibrium

$$\left(u^{*},v^{*},w^{*}\right),\;u^{*}>0,\;v^{*}>0,\;w^{*}>0,$$

where

(A8) $$v^{*}=1-u^{*},\;w^{*}=\frac{u^{*}\left[\alpha +\gamma \left(1-u^{*}\right)\right]}{l}.$$

### Appendix B.1. Linearization About Coexistence

Let

$$u=u^{*}+\tilde{u},\;v=v^{*}+\tilde{v},\;w=w^{*}+\tilde{w}.$$

Keeping only first-order terms gives

(A9) $$\begin{array}{cc} {\tilde{u}}_{t}\; & =d_{1}\Delta \tilde{u}+\left(1-2u^{*}-v^{*}\right)\tilde{u}-u^{*}\tilde{v},\\ {\tilde{v}}_{t}\; & =d_{2}\Delta \tilde{v}-\xi v^{*}\Delta \tilde{w}-\beta v^{*}\tilde{u}-\left(\delta +\beta u^{*}\right)\tilde{v}+\frac{\sigma_{1}}{{\left(1+w^{*}\right)}^{2}}\tilde{w},\\ {\tilde{w}}_{t}\; & =d_{3}\Delta \tilde{w}+\left(\alpha +\gamma v^{*}\right)\tilde{u}+\gamma u^{*}\tilde{v}-l\tilde{w}. \end{array}$$

Since $v^{*}=1-u^{*}$, we have $1-2u^{*}-v^{*}=-u^{*}$, so the first equation becomes

$${\tilde{u}}_{t}=d_{1}\Delta \tilde{u}-u^{*}\tilde{u}-u^{*}\tilde{v}.$$

The sign of the chemotaxis term is important. Since the chemotactic contribution is −ξ∇·(v∇w), its linearization at the homogeneous equilibrium is

$$-\xi \nabla \cdot \left(v^{*}\nabla \tilde{w}\right)=-\xi v^{*}\Delta \tilde{w}.$$

After projection onto a Neumann eigenmode, this term becomes a positive (v,w)-coupling, as shown below.

### Appendix B.2. Projection onto Neumann Modes

Let ${\left\{\phi_{k}\right\}}_{k=0}^{\infty }$ be the Neumann Laplacian eigenfunctions,

$$-\Delta \phi_{k}=\mu_{k}\phi_{k},\;\partial_{n}\phi_{k}=0\;\mathrm{on}\;\partial \Omega ,\;0=\mu_{0}<\mu_{1}\leq \mu_{2}\leq \ldots .$$

For a single mode, write

$$\tilde{u}\left(t,x\right)=U_{k}\left(t\right)\phi_{k}\left(x\right),\;\tilde{v}\left(t,x\right)=V_{k}\left(t\right)\phi_{k}\left(x\right),\;\tilde{w}\left(t,x\right)=W_{k}\left(t\right)\phi_{k}\left(x\right).$$

Since $\Delta \phi_{k}=-\mu_{k}\phi_{k}$, the modal amplitudes satisfy

$$\frac{d}{dt}\left(\begin{array}{c} U_{k}\\ V_{k}\\ W_{k} \end{array}\right)=M_{k}\left(\begin{array}{c} U_{k}\\ V_{k}\\ W_{k} \end{array}\right),$$

where

(A10) $$M_{k}=\left(\begin{array}{ccc} -u^{*}-d_{1}\mu_{k} & -u^{*} & 0\\ -\beta v^{*} & -\left(\delta +\beta u^{*}\right)-d_{2}\mu_{k} & \frac{\sigma_{1}}{{\left(1+w^{*}\right)}^{2}}+\xi v^{*}\mu_{k}\\ \alpha +\gamma v^{*} & \gamma u^{*} & -l-d_{3}\mu_{k} \end{array}\right).$$

The positive contribution $+\xi v^{*}\mu_{k}$ in the (2,3)-entry follows from

$$-\xi v^{*}\Delta \tilde{w}=-\xi v^{*}\left(-\mu_{k}W_{k}\phi_{k}\right)=\xi v^{*}\mu_{k}W_{k}\phi_{k}.$$

Thus chemotaxis has no effect on the homogeneous mode through this term because $\mu_{0}=0$, but it can destabilize finite spatial modes with $\mu_{k}>0$. This is the matrix (38) used in the main text.

### Appendix B.3. Characteristic Polynomial

For compactness define

(A11) $$A_{k}=u^{*}+d_{1}\mu_{k},\;B_{k}=\delta +\beta u^{*}+d_{2}\mu_{k},\;C_{k}=l+d_{3}\mu_{k},$$

and

(A12) $$p=u^{*},\;q=\beta v^{*},\;r_{k}=\frac{\sigma_{1}}{{\left(1+w^{*}\right)}^{2}}+\xi v^{*}\mu_{k},\;s=\alpha +\gamma v^{*},\;t=\gamma u^{*}.$$

Then (A10) can be written as

(A13) $$M_{k}=\left(\begin{array}{ccc} -A_{k} & -p & 0\\ -q & -B_{k} & r_{k}\\ s & t & -C_{k} \end{array}\right).$$

The characteristic polynomial is $P_{k}\left(\lambda \right)=\det\left(\lambda I-M_{k}\right)$. Since

$$\lambda I-M_{k}=\left(\begin{array}{ccc} \lambda +A_{k} & p & 0\\ q & \lambda +B_{k} & -r_{k}\\ -s & -t & \lambda +C_{k} \end{array}\right),$$

expansion along the first row gives

$$P_{k}\left(\lambda \right)=\left(\lambda +A_{k}\right)\left[\left(\lambda +B_{k}\right)\left(\lambda +C_{k}\right)-r_{k}t\right]-p\left[q\left(\lambda +C_{k}\right)-r_{k}s\right].$$

Therefore

$$P_{k}\left(\lambda \right)=\lambda^{3}+a_{1}\left(k\right)\lambda^{2}+a_{2}\left(k\right)\lambda +a_{3}\left(k\right),$$

where

(A14) $$a_{1}\left(k\right)=A_{k}+B_{k}+C_{k},$$

(A15) $$a_{2}\left(k\right)=A_{k}B_{k}+A_{k}C_{k}+B_{k}C_{k}-r_{k}t-pq,$$

(A16) $$a_{3}\left(k\right)=A_{k}B_{k}C_{k}-A_{k}r_{k}t-pqC_{k}+pr_{k}s.$$

Equivalently,

(A17) $$a_{3}\left(k\right)=A_{k}B_{k}C_{k}-C_{k}pq+r_{k}\left(ps-A_{k}t\right).$$

### Appendix B.4. Routh–Hurwitz Conditions

The mode *k* is linearly stable if and only if all roots of $P_{k}\left(\lambda \right)=\lambda^{3}+a_{1}\left(k\right)\lambda^{2}+a_{2}\left(k\right)\lambda +a_{3}\left(k\right)$ lie in the open left half-plane. By the Routh–Hurwitz criterion for a monic cubic, this holds if and only if

(A18) $$a_{1}\left(k\right)>0,\;a_{2}\left(k\right)>0,\;a_{3}\left(k\right)>0,\;a_{1}\left(k\right)a_{2}\left(k\right)>a_{3}\left(k\right).$$

Since $A_{k},B_{k},C_{k}>0$, we always have $a_{1}\left(k\right)>0$. The remaining inequalities encode the competition between diffusive damping, reaction damping, chemokine recruitment, and chemotactic amplification.

### Appendix B.5. Chemotactic Contribution to the Constant Term

The dependence of $a_{3}\left(k\right)$ on the chemotactic sensitivity ξ is especially important. Since $r_{k}=\sigma_{1}/{\left(1+w^{*}\right)}^{2}+\xi v^{*}\mu_{k}$, we can rewrite (A17) as

(A19) $$a_{3}\left(k;\xi \right)=\Lambda_{k}+\xi v^{*}\mu_{k}\left(ps-A_{k}t\right),$$

where

(A20) $$\Lambda_{k}=A_{k}B_{k}C_{k}-C_{k}pq+\frac{\sigma_{1}}{{\left(1+w^{*}\right)}^{2}}\left(ps-A_{k}t\right).$$

Equivalently,

(A21) $$a_{3}\left(k;\xi \right)=\Lambda_{k}-\xi v^{*}\mu_{k}\left(A_{k}t-ps\right).$$

Thus, if

(A22) $$A_{k}t-ps>0,$$

then increasing ξ decreases $a_{3}\left(k;\xi \right)$. In this case, if $\Lambda_{k}>0$, the *k*-th mode violates the Routh–Hurwitz condition $a_{3}\left(k\right)>0$ whenever

(A23) $$\xi >\xi_{k}^{*}:=\frac{\Lambda_{k}}{v^{*}\mu_{k}\left(A_{k}t-ps\right)}.$$

If $\Lambda_{k}\leq 0$, then $a_{3}\left(k;\xi \right)\leq 0$ already at ξ=0, so the mode is unstable before chemotaxis is increased. Hence (A23) is a useful chemotactic threshold only in the regime $\Lambda_{k}>0,\;A_{k}t-ps>0$. The first instability generated through this mechanism occurs at

(A24) $$\xi_{c}=\min_{\begin{array}{c} k\geq 1\\ \Lambda_{k}>0,\;A_{k}t-ps>0 \end{array}}\xi_{k}^{*}.$$

The restriction k≥1 is necessary because the homogeneous mode has $\mu_{0}=0$, and the chemotactic contribution $\xi v^{*}\mu_{k}$ vanishes for k=0.

### Appendix B.6. Alternative Instability Through the Determinant Condition

The condition $a_{3}\left(k\right)>0$ is only one of the Routh–Hurwitz inequalities. A mode may also lose stability through $a_{2}\left(k\right)=0$ or through $a_{1}\left(k\right)a_{2}\left(k\right)-a_{3}\left(k\right)=0$. The latter corresponds to a Hopf-type crossing for the modal ODE system when the remaining Routh–Hurwitz inequalities hold. Therefore, in numerical stability maps it is safest to compute

ωk(ξ)=maxjReλj(Mk)

directly and define the onset by $\max_{k\geq 1}\omega_{k}\left(\xi \right)=0$. The explicit threshold (A23) should be interpreted as an analytical sufficient destabilization criterion through the constant term $a_{3}\left(k\right)$, not as the only possible instability mechanism. Indeed, for the parameter set of Section 7 one has $A_{k}t-ps<0$ in the relevant wavenumber band, so the constant-term mechanism is inactive and the onset is the Hopf-type crossing.
